# Supplementary material for: Leave the world(view) behind, but keep the words: The effect of conspiracism on writing
Source: PLoS One. 2026 Apr 29;21(4):e0346496. doi: 10.1371/journal.pone.0346496 (PMC13127973; doi:10.1371/journal.pone.0346496)
Supplement: S1 File — (PDF) [file pone.0346496.s001.pdf]

SUPPORTING INFORMATION

Leave the world(view) behind, but keep the words: The effect of conspiracism on writing

Alessandro Miani[1,2*], Ines Adornetti[3], Daniela Altavilla[3], Valentina Deriu[3], Alessandra Chiera[3], Francesco Ferretti[3]

1 School of Psychological Science, University of Bristol, United Kingdom
2 Department of Psychology, University of Fribourg, Switzerland
3 Cosmic Lab, Department of Philosophy, Communication and Performing Arts, Roma Tre University, Rome, Italy

* alessandro.miani@bristol.ac.uk

S1 Prompts

Prompt S1. Prompt to write about the film

```
Ora scrivi qui sotto la tua interpretazione del contenuto del film.  Attenzione:  è
il significato che tu hai dato al film che ti chiediamo di riportare, non un riassunto
della trama.  Non c'è una risposta giusta o sbagliata.  Ti chiediamo solo di scrivere
almeno (minimo) fino alla fine di questo foglio (puoi usare anche l'altra facciata del
foglio nel caso questa non dovesse bastarti).
```

English translation: Now write your interpretation of the content of the film below. Please note: we are asking for the meaning you gave to the film, not a summary of the plot. There is no right or wrong answer. We only ask that you write at least until the end of this sheet (you may also use the other side of the sheet if this is not enough).

Prompt S2. Evaluate Italian essays

```
You are given an essay written in Italian.  Please evaluate the extent to which the
essay presents a conspiratorial narrative.  A conspiratorial narrative is one that
suggests the public is being pervasively lied to regarding some aspect(s) of reality,
to allow some group(s) to enact a harmful, self-serving agenda.  Focus solely on the
content of the narrative, excluding any meta-comments such as "Secondo me, questo film
può essere interpretato..".  Provide an integer score between 0 and 10, where 0 means
no conspiratorial narrative and 10 means a very strong conspiratorial narrative.  Do
not provide any explanation, just the score.  The essay is as follows < ESSAY >
```

Prompt S3. Translate conspiracy dictionary from English to Italian

```
I give you a list of 100 English lemmatized words related to conspiratorial language.
Please translate each word into Italian.  The output must be ONLY a table with
two columns (which I will import into R, so consistency is crucial):  the first
column should contain the English words, and the second column should contain the
corresponding Italian translations.  If there is more than one possible translation,
include all translations, separated by a comma.  Here is the list of words, separated
by comma:  < LIST OF LEMMAS >
```

Prompt S4. Generate corpus of High vs Low syntactic complexity

```
I need a corpus to test a measure of syntactic sophistication.  Please create 50
documents, split evenly into two groups:  25 documents labeled L_1:25 to represent
low complexity, and 25 documents labeled H_1:25 to represent high complexity.  Each
document must contain at least two sentences, and the high-complexity documents should
include more intricate syntactic structures, than their low-complexity counterparts.
Please return the corpus as an R dataframe with two columns:  doc_id, containing the
document labels, and document, containing the text for each document.  Ensure that
each pair of documents (L_n, H_n) covers a comparable topic but differs in syntactic
complexity, so that the high-complexity entries are recognizably more complex than
their matching low-complexity entries.
```

Prompt S5. Extract lexical sophistication norms in Italian

```
I will provide a list of 100 lemmatized Italian words, each followed by its part of
speech (e.g., "mamma_NOUN" or "andare_VERB"). Your task is to assess the complexity of
each word in a way similar to age of acquisition norms, focusing on how sophisticated
each word is for a native Italian speaker.  Please rate each word on a scale from 0
to 100, where 0 indicates the lowest level of complexity (for words typically acquired
at a very young age, such as "mamma" or "pappa") and 100 represents the highest level
of complexity (for words typically learned later or those considered jargon, such as
"sifilide," "cherosene," or "nevrotico").  If you come across an unfamiliar word, make
an educated guess regarding its complexity.  The output must be ONLY a table with two
columns (which I will import in R, so consistency is crucial):  the first listing the
words and the second containing their corresponding complexity ratings.  Here is the
list of words, separated by comma:  < LIST OF LEMMAS >
```

S2 The Italian dictionary of conspiracy theories

Table S1. The Italian dictionary of conspiracy theories

5g, aborto, abuso, adorazione, affermazione, agenda, agente, alieno, alluminio, america, americano, ammettere, anima, anti, anticristo, apertamente, arma, arresto, articolo, assalto, assassinio, asterisco, attacco, autismo, avviso_legale, aziendale, banca, banchiere, bandiera, bibbia, biblico, bilderberg, bomba, bufala, bugia, burattino, bush, cabala, cancello, cartello, catastrofico, cattivo, cattolico, censore, censura, cespuglio, chemtrails, chiesa, chimico, cia, citazione, cittadino, civiltà, clinton, coincidenza, collasso, collegamento, colpo_di_stato, comunismo, comunista, connessione, controllo, corporazione, corrente_principale, corrotto, corruzione, coscienza, cospirazione, costituzione, criminale, crimine, cristianesimo, cristiano, culto, debito, deliberatamente, democratico, democrazia, demone, demonico, denaro, depopolazione, di_sinistra, diavolo, difesa, diritto_d'autore, discernimento, disinformazione, distruggere, distruzione, documento, dollaro, dottore, dovrà, ebreo, ecc, élite, ellissi, entità, epstein, esporre, essere, establishment, evidenza, faal, falso, farmaceutico, fascista, fatto, fattura, fbi, fino_a, fitts, folle, fonte, forza, frode, genocidio, geoingegneria, george, gesuita, giornalista, globale, globalista, globalisti, google, governo, guerra, hillary, hitler, ignorare, illegale, illuminati, impero, in_realtà, incidente, indagine, inferno, infiltrare, influenza, informatore, infowars, ingannare, inganno, ingegnere, innocente, intelligenza, internet, intero, invasione, investigativo, kennedy, lavaggio_del_cervello, legge, letteralmente, libertà, libro, lucifero, mafia, male, manipolare, manipolazione, marziale, massa, massiccio, massone, massoneria, massonico, matrice, mente, menzionare, mercurio, mezzo, militare, mk, mondo, monsanto, morte, msm, mueller, narrazione, nascondere, nato, nazista, nazisti, nemico, niente, noi, nsa, nucleare, nutrire, nwo, obama, occidentale, occulto, odio, ogm, omicidio, omosessuale, operativo, operazione, ordine, oro, paradiso, parola, patriota, peccato, pedofilo, per_favore, perché, petrolio, pfc, pianeta, piramide, polizia, potente, potere, prigione, profezia, profitto, profondo, propaganda, prova, punto_esclamativo (!), punto_interrogativo (?), putin, questione, qui, reale, realtà, regime, regno, religione, ricchezza, riserva, risvegliare, rituale, rivelazione, rivendicare, rockefeller, romano, rothschild, rovesciare, rubare, sacrificio, satana, satanico, schiavitù, schiavo, scopo, screditare, segreto, seme, sesso, shipp, signore, simbolismo, simbolo, sionista, sito_web, socialista, società, sopra, sorcha, soros, sotto, spia, spirito, spirituale, spray, su, suicidio, suppostamente, teoria, teorico, terra, terrore, terrorismo, terrorista, tirannia, tlb, tortura, tossico, trilione, trump, tutto, uccidere, ufo, umanità, vaccinazione, vaccino, valuta, vangelo, veleno, verità, vero, video, youtube.

S3 An alternative evaluation using LLMs

We asked Claude to evaluate essays using a multidimensional approach, relying on dimensions previously identified in conspiratorial discourse [1, 2]. These dimensions are:

- *Social relevance* (to what extent does the essay reference themes of collective or societal importance, e.g., institutions, media, power, or the public)
- *Deception* (to what extent does the essay suggest that the official or mainstream version of events is false or misleading)
- *Malevolence* (to what extent does the essay attribute harmful or malicious intent to individuals, groups, or institutions)
- *Metacommunicative denial* (to what extent does the essay reject facts, evidence, or counterarguments by linking them to untrustworthy mainstream or official sources, rather than responding to their content)
- *Epistemic distancing* (to what extent does the essay use linguistic distancing (e.g., scare quotes or hedging) from institutional terms such as truth, proof, or facts)
- and *Truth-seeking stance* (to what extent does the author position themselves as a "truth-seeker" in opposition to misled or manipulated individuals, often implying moral or intellectual superiority)

Each dimension was rated for each essay on a score from 0 to 10 based on its presence (see Prompt S6). All dimensions were averaged and aggregated into a unique score for each essay. In Table S2, we show the correlations of the multidimensional score (aggregated as well as individual dimensions) with conspiracism, conspiratorial narrative scores based on the preregistered LLM assessments, and conspiratorial lexicon scores based on dictionary assessments.

Prompt S6. Evaluate Italian essays (using three dimensions)

```
You will receive an essay written in Italian by a study participant.  Your task
is to evaluate the presence of conspiratorial discourse based on the following
six dimensions:  a) Social relevance - To what extent does the essay reference
themes of collective or societal importance (e.g., institutions, media, power,
or the public)?  b) Deception - To what extent does the essay suggest that the
official or mainstream version of events is false or misleading?  c) Malevolence
- To what extent does the essay attribute harmful or malicious intent to
individuals, groups, or institutions?  d) Metacommunicative denial - To what
extent does the essay reject facts, evidence, or counterarguments by linking
them to untrustworthy mainstream or official sources, rather than responding
to their content?  e) Epistemic distancing - To what extent does the essay use
linguistic distancing (e.g., scare quotes or hedging) from institutional terms
such as 'truth' [verità], 'proof' [prove], or 'facts' [fatti]?  f) Truth-seeking
stance - To what extent does the author position themselves as a 'truth-seeker'
in opposition to misled or manipulated individuals, often implying moral or
intellectual superiority?  For each dimension, assign an integer score from 0
(Not present) to 10 (Extremely present).  The output must be a single-row table
(in plain text format) with exactly six columns, named:  social, deception,
malevolence, denial, distancing, and truth_seeking (in that order).  Do not
include any explanation or extra commentary.  Focus only on the content of
the essay.  Ignore any meta-comments or task-related notes written by the
participant.  The essay to evaluate is:  < ESSAY >
```

Table S2. Pearson correlation coefficients between participants' conspiracism, essays' narrative and lexical conspiracy levels, and alternative metrics of essay conspiratorial content

MD: multidimension assessment	conspiracism	CT narrative	CT lexicon
MD aggregated	-.01	.67	.28
MD social	-.04	.50	.18
MD deception	.03	.75	.26
MD malevolence	-.02	.64	.27
MD denial	-.02	.65	.23
MD distancing	.02	.43	.22
MD truth seeking	.01	.54	.29

Note. $N = 385$. Conspiracism scores were obtained by rescaling CMS and GCB responses to a 0–1 range. *CT Narrative*: Conspiratorial narrative scores based on our main LLM assessments (see main text); *CT Lexicon*: Conspiratorial lexicon scores based on dictionary assessments; *MD (aggregated)*: Conspiratorial narrative scores based on multidimension LLM assessments (aggregated dimensions), see Prompt S6.

S4 Textual quality as moderator of Essays' rating

To examine whether textual quality influences how different rating methods detect conspiracy content, we investigated the moderating role of linguistic sophistication on the relationship between participants' conspiracism and essay ratings. We hypothesized that LLM-based classifications would be particularly susceptible to textual quality effects, potentially confounding content assessment with writing proficiency. As preregistered, we initially assessed essay conspiracism using ChatGPT. However, subsequent exploration revealed that Claude's ratings showed higher correlation with human manual annotations ($r = .701$ vs $r = .676$, see section "*Assessing conspiratorial narrative using LLMs*"). While the main text reports analyses using Claude ratings for their superior validity, we present results for both LLMs here for transparency and methodological completeness.

We conducted nine moderated regression analyses, systematically crossing three rating methods (ChatGPT, Claude, dictionary) with three textual quality measures (syntactic sophistication, lexical sophistication, cohesion). Each model predicted essay conspiracism scores from participant conspiracism, textual quality, and their interaction. All predictors were z-standardized to facilitate interpretation and comparison across models. All models followed the general formula:

$$Y = \beta_1 Q + \beta_2 M + \beta_3 (Q \times M) \quad (1)$$

where Y represents the conspiracy rating method (ChatGPT for models A, D, G; Claude for models B, E, H; dictionary for models C, F, I), Q represents textual quality measures (syntactic sophistication for models A–C; lexical sophistication for models D–F; cohesion for models G–I), and M represents conspiracy mentality across all models. Figure S1 displays the regression slopes, while Table S3 reports standardized coefficients.

To quantify moderation strength, we calculated slope ranges as twice the absolute value of each interaction coefficient ($2 \times |\beta_3|$). This metric represents the total difference in conspiracy mentality's slope when comparing texts at -1 SD versus $+1$ SD of textual quality. Larger slope ranges indicate stronger moderating effects, meaning textual quality more substantially alters the relationship between conspiracy mentality and essay ratings. The dictionary-based scoring showed the smallest average slope range (.074), followed by Claude (.097) and ChatGPT (.118), potentially indicating less influence of textual quality on conspiracy assessment in essays via dictionary as compared to LLMs. LLM-based rating systems, particularly ChatGPT, are more vulnerable to confounding effects of writing quality when evaluating conspiracy content, whereas dictionary-based approaches provide more stable content assessment independent of linguistic sophistication.

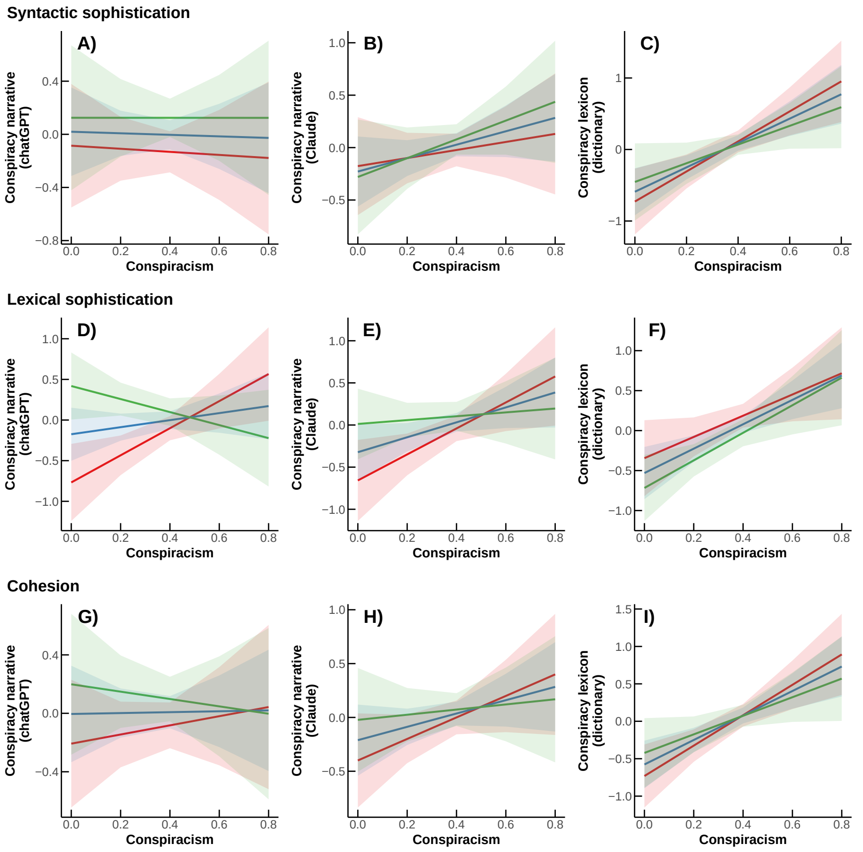

Figure S1. Essays' conspiracy assessment by participants' conspiracism level moderated by textual quality. Predictions are shown for texts that are 1 SD below the sample mean (red, "low" quality), at the mean (blue), and 1 SD above the mean (green, "high" quality) on the relevant textual quality measure (syntactic sophistication, lexical sophistication, and cohesion). Shaded area represents the 95% confidence intervals.

Table S3. Standardized regression coefficients for moderated regression analyses examining textual quality effects on conspiracy content detection across models shown in Figure S1

Model	term	β (SE)	$P(>\lvert t\rvert)$	range
A) **ChatGPT** ~ **Syntactic Sophistication**	M	-.007 (.052)	.899	
	Q	.125 (.055)	**.023**	
	M:Q	.006 (.054)	.905	.013
B) **Claude** ~ **Syntactic Sophistication**	M	.072 (.052)	.165	
	Q	.038 (.055)	.490	
	M:Q	.029 (.054)	.596	.058
C) **Dictionary** ~ **Syntactic Sophistication**	M	.192 (.051)	< **.001**	
	Q	-.002 (.054)	.969	
	M:Q	-.045 (.053)	.404	.089
D) **ChatGPT** ~ **Lexical Sophistication**	M	.049 (.051)	.337	
	Q	.160 (.051)	**.002**	
	M:Q	-.139 (.049)	**.005**	.278
E) **Claude** ~ **Lexical Sophistication**	M	.100 (.051)	.053	
	Q	.105 (.052)	**.045**	
	M:Q	-.074 (.050)	.141	.148
F) **Dictionary** ~ **Lexical Sophistication**	M	.172 (.051)	**.001**	
	Q	-.117 (.051)	**.024**	
	M:Q	.022 (.049)	.652	.045
G) **ChatGPT** ~ **Cohesion**	M	.003 (.051)	.946	
	Q	.104 (.051)	**.043**	
	M:Q	-.032 (.049)	.519	.064
H) **Claude** ~ **Cohesion**	M	.070 (.051)	.176	
	Q	.055 (.051)	.282	
	M:Q	-.043 (.049)	.385	.085
I) **Dictionary** ~ **Cohesion**	M	.184 (.049)	< **.001**	
	Q	.016 (.049)	.750	
	M:Q	-.044 (.047)	.349	.089

Each model predicts conspiracy ratings (via **ChatGPT**, **Claude**, and **Dictionary**) from conspiracy mentality (M), textual quality measures (Q: Lexical Sophistication, Syntactic Sophistication, and Cohesion), and their interaction (M:Q). Models A–C examine syntactic sophistication, models D–F examine lexical sophistication, and models G–I examine cohesion as moderators. The range column shows twice the absolute value of the interaction coefficient, representing the total difference in conspiracy mentality's slope between texts at −1 SD versus +1 SD of textual quality. Larger ranges indicate stronger moderation effects. Standard errors (SE) are shown in parentheses. Significant coefficients ($p < .05$) are shown in bold.

S5 Exploratory psychological variables

For exploratory purposes, we collected data on attachment style and pathological narcissism. This decision was guided by previous research highlighting associations between conspiracism and narcissism [3–8] as well as between conspiracism and attachment dimensions [8–10].

The Pathological Narcissism Inventory (PNI [11]; Italian version by [12]), a 52-item self-report measure, was used to assess 7 dimensions of pathological narcissism refer to narcissistic grandiosity (exploitativeness, grandiose fantasy, sacrificial self-enhancement) and narcissistic vulnerability (contingent self-esteem, entitlement anger, hiding the self, devaluation), which are rated on a 6-point scale ranging from 1 (“strongly disagree”) to 6 (“strongly agree”).

The Attachment Style Questionnaire (ASQ [13]; Italian version by [14]), a 40-item self-report measure, was used to assess the 5 dimensions of attachment: confidence which refer to secure attachment; concern about relationships and need for approval which refer to the anxious attachment; discomfort with intimacy and secondary relationships which refer to avoidant attachment, which are rated on a 6-point scale ranging from 1 (“strongly disagree”) to 6 (“strongly agree”).

Correlation coefficients between conspiracy-related variables and exploratory psychological variables are shown in Table S4 and Table S5.

Table S4. Correlation coefficients between conspiracy-related variables and exploratory psychological variables in Study 1

	**CMS**			**Essays**					
	Total	(Idea)	(Skept)	CTN	CTL	SL	SS	CH	ML
Conspiratorial Narrative	.06	.06	.04			.08	.03	.06	-.02
Conspiratorial Lexicon	.18**	.16**	.14*			-.15**	.02	.05	.20***
PNI-Exploitativeness	.11.	.09	.10	-.01	-.00	.12*	.05	-.05	.08
PNI-Grandiose fantasy	.22***	.19**	.21***	-.08	-.01	.04	.11.	.13*	.05
PNI-Sacrificial self-enhancement	.21***	.22***	.11.	-.08	.06	-.06	-.07	-.01	.07
PNI-Total Narcissistic grandiosity	.25***	.23***	.20***	-.09	.02	.04	.05	.05	.08
PNI-Contingent self-esteem	.09	.08	.07	-.03	.08	-.09	-.04	.06	.06
PNI-Entitlement anger	.25***	.24***	.16**	-.02	.14*	-.02	.00	.03	.13*
PNI-Hiding the self	.04	.00	.10.	-.00	.05	.07	-.16**	-.05	-.01
PNI-Devaluation	.18**	.20***	.06	.01	.04	-.01	-.08	.02	.02
PNI-Total Narcissistic vulnerability	.16**	.16**	.12.	-.02	.10	-.03	-.08	.03	.07
ASQ-Confidence	.11.	.13*	.02	.05	-.01	-.01	-.03	-.06	.04
ASQ-Discomfort with intimacy	.04	.00	.12*	-.01	-.02	.05	-.05	-.00	-.01
ASQ-Secondary relationships	.02	.04	-.02	.01	-.05	.04	.02	.06	-.05
ASQ-Concern about relationships	.11.	.06	.18**	-.04	.01	-.00	.03	.06	.12*
ASQ-Need for approval	-.04	-.06	.02	-.05	-.02	-.08	-.07	.07	.02

Note: *$p < .05$, **$p < .01$, ***$p < .001$. PNI = Pathological Narcissism Inventory; ASQ = Attachment Style Questionnaire; CTN/CTL = essays' conspiratorial narrative/lexicon; CMS = Conspiracy Mentality Scale (Idea = ideation; skept = skepticism); SL/SS = lexical/syntactic sophistication, CH = cohesion, ML = megalalia

Table S5. Correlation coefficients between conspiracy-related variables and exploratory psychological variables in Study 1

	**GCB**						**Essays**					
	Total	(1)	(2)	(3)	(4)	(5)	CTN	CTL	SL	SS	CH	ML
Conspiratorial Narrative	.11	.22*	.09	-.09	.13	.08			.15	.14	.04	-.18.
Conspiratorial Lexicon	.23*	.19.	.23*	.03	.19.	.26**			-.12	-.02	-.06	-.14
PNI-Exploitativeness	.07	-.07	-.07	.29**	.03	.10	-.04	.08	.14	-.02	-.23*	.14
PNI-Grandiose fantasy	-.02	-.02	-.05	.01	-.06	.08	-.22*	-.14	.03	-.14	-.16	.07
PNI-Sacrificial self-enhancement	-.02	.02	-.05	-.06	.00	.03	-.01	-.14	.04	.03	.02	-.09
PNI-Total Narcissistic grandiosity	.01	-.03	-.08	.10	-.02	.09	-.14	-.10	.09	-.07	-.16	.05
PNI-Contingent self-esteem	.01	.06	.02	-.07	-.10	.14	-.00	-.07	-.02	.01	-.06	-.01
PNI-Entitlement anger	.01	-.01	-.02	-.05	.03	.08	-.12	-.13	-.12	.03	-.07	.14
PNI-Hiding the self	.04	.05	-.04	.01	-.02	.20*	-.10	-.05	-.08	-.05	-.11	.01
PNI-Devaluation	-.00	.06	-.08	-.06	.00	.08	.06	-.16	.03	.07	-.01	.01
PNI-Total Narcissistic vulnerability	.02	.05	-.04	-.06	-.03	.16	-.05	-.13	-.05	.01	-.08	.04
ASQ-Confidence	-.07	-.14	.00	-.07	.01	-.10	.11	.17.	.04	-.14	-.12	-.05
ASQ-Discomfort with intimacy	-.07	-.02	-.06	-.11	-.16	.09	-.09	.01	-.06	.06	-.01	.04
ASQ-Secondary relationships	-.08	-.15	.06	.02	-.19.	-.01	-.31**	-.07	.11	.00	-.01	.01
ASQ-Concern about relationships	.03	.14	-.06	-.01	-.03	.11	-.00	-.08	.02	-.08	-.07	-.13
ASQ-Need for approval	-.06	-.00	.03	-.17.	-.12	.06	-.12	-.00	-.07	-.14	-.03	-.08

Note: $^{*}p < .05$, $^{**}p < .01$, $^{***}p < .001$. PNI = Pathological Narcissism Inventory; ASQ = Attachment Style Questionnaire; GCB = Generic Conspiratorial Belief Scale; GCB factors: (1) = Government malfeasance; (2) = Malevolent global conspiracies; (3) = Extraterrestrial cover-up; (4) = Personal wellbeing; (5) = Control of information; CTN/CTL = essays' conspiratorial narrative/lexicon; SL/SS = lexical/syntactic sophistication, CH = cohesion, ML = megalalia

S6 Correlations with LIWC variables

Table S6. Correlation coefficients between conspiracy-related variables and LIWC dictionaries

**1. Conspiracism**		**2. Narrative**		**3. Lexicon**	
variable	*r*	variable	*r*	variable	*r*
riempiti	.13	Musica	.13	Discrep	.34***
Sotto	.12	Loro_Ver	.11	Essere	.32***
Passato	.12	Condizio	.11	Noi_Verb	.27***
Negazio	.12	Dormire	.11	pronomi	.26***
parolac	.12	possib	.10	Condizio	.26***
Mangiare	.10	Umano	-.11	Present	.26***
Cura_cor	.10	Altri	-.12	Noi	.25***
Ottimis	-.10	Famigl	-.12	Inclusi	.21**
Affett	-.10	amici	-.12	Se	.21**
Prepos	-.14	TV_it	-.13	Esclusi	.20**
BigWords	-.14	Social	-.14	Io_Ver	.18*
				possib	.17
				Mec_Cog	.15
				Morte	.15
				Passato	.13
				Loro_Ver	.13
				Sopra	.13
				Mangiare	.11
				Io	.10
				Certez	.10
				Sen_Pos	-.11
				Numero	-.13
				Ottimis	-.14
				Prepos	-.15
				Casa	-.16
				Social	-.17
				BigWords	-.18*
				Umano	-.18*
				Transiti	-.20**
				Altri	-.24***
				Articol	-.25***
				Famigl	-.31***

Note. Correlations between LIWC dictionaries and (1) conspiracism, (2) conspiracy narratives assessed by ChatGPT, and (3) conspiracy narratives assessed via the conspiracy dictionary. $^{*}p < .05$, $^{**}p < .01$, $^{***}p < .001$, Bonferroni corrected for 89 tests. Only dictionaries that were significant at $p < .05$ before correction are reported. **Affett**: *Affect*; **Altri**: *Other*; **amici**: *Friends*; **Articol**: *Articles*; **BigWords**: *Words > 6 letters*; **Casa**: *Home*; **Certez**: *Certainty*; **Condizio**: *Conditional*; **Cura_cor**: *Health/Body Care*; **Dic**: *Dictionary words*; **Discrep**: *Discrepancy*; **Esclusi**: *Exclusion*; **Essere**: *Be verbs*; **Famigl**: *Family*; **Inclusi**: *Inclusion*; **Io**: *I*; **Io_Ver**: *I verb*; **Loro_Ver**: *They verb*; **Lui_lei**: *Third-person singular*; **Mangiare**: *Eating*; **Mec_Cog**: *Cognitive Processes*; **Morte**: *Death*; **Musica**: *Music*; **Negazio**: *Negations*; **Noi**: *We*; **Noi_Verb**: *We verb*; **Numero**: *Numbers*; **Ottimis**: *Optimism*; **parolac**: *Swear Words*; **Passato**: *Past*; **Period**: *Period*; **possib**: *Tentative*; **Prepos**: *Prepositions*; **Present**: *Present*; **pronomi**: *Pronouns*; **riempiti**: *Fillers*; **Se**: *Myself, Ourselves*; **Sen_Pos**: *Positive Sentiment*; **Sesso**: *Sexual*; **Social**: *Social Processes*; **Sopra**: *Up*; **Sotto**: *Down*; **Svago**: *Leisure*; **Transiti**: *Verbal Conjugations*; **TV_it**: *TV/movies*; **Umano**: *Human.*

References

1. Cedric Deschrijver. On the metapragmatics of conspiracy theory: Scepticism and epistemological debates in online conspiracy comments. *Journal of Pragmatics*, 182:310–321, 2021. ISSN 0378-2166. doi: 10.1016/j.pragma.2021.02.010.

2. Karen M. Douglas, Joseph E. Uscinski, Robbie M. Sutton, Aleksandra Cichocka, Turkay Nefes, Chee Siang Ang, and Farzin Deravi. Understanding conspiracy theories. *Political Psychology*, 40 (S1):3–35, 2019. ISSN 0162-895X, 1467-9221. doi: 10.1111/pops.12568.

3. Tylor J. Cosgrove and Christopher P. Murphy. Narcissistic susceptibility to conspiracy beliefs exaggerated by education, reduced by cognitive reflection. *Frontiers in Psychology*, 14, 2023. ISSN 1664-1078. doi: 10.3389/fpsyg.2023.1164725.

4. Aleksandra Cichocka, Marta Marchlewska, and Agnieszka Golec de Zavala. Does self-love or self-hate predict conspiracy beliefs? Narcissism, self-esteem, and the endorsement of conspiracy theories. *Social Psychological and Personality Science*, 7(2):157–166, 2016.

5. Aleksandra Cichocka, Marta Marchlewska, and Mikey Biddlestone. Why do narcissists find conspiracy theories so appealing? *Current Opinion in Psychology*, 47:101386, 2022. ISSN 2352-250X. doi: 10.1016/j.copsyc.2022.101386.

6. Aleksander B. Gundersen, Sander van der Linden, Jan Piasecki, Rafa Rygua, Karolina Noworyta, and Jonas R. Kunst. Predicting misinformation beliefs across four countries: The role of narcissism, conspiracy mentality, social trust, and perceptions of unsafe neighborhoods. *Journal of Social and Political Psychology*, 12(2): 265–283, 2024. ISSN 2195-3325. doi: 10.5964/jspp.13385.

7. Cameron S. Kay. The targets of all treachery: Delusional ideation, paranoia, and the need for uniqueness as mediators between two forms of narcissism and conspiracy beliefs. *Journal of Research in Personality*, 93:104128, 2021. ISSN 0092-6566. doi: 10.1016/j.jrp.2021.104128.

8. Emiliano Loria and Cristina Meini. Uncertainty, attachment, and narcissism, but most of all vulnerability: the perfect recipe for conspiracy therapy. *Rivista Italiana di Filosofia del Linguaggio*, 2023. ISSN 20366728. doi: 10.4396/2022SFL14.

9. Luigi Leone, Mauro Giacomantonio, Riccardo Williams, and Desirée Michetti. Avoidant attachment style and conspiracy ideation. *Personality and Individual Differences*, 134:329–336, 2018. ISSN 0191-8869. doi: 10.1016/j.paid.2018.06.043.

10. Ricky Green and Karen M. Douglas. Anxious attachment and belief in conspiracy theories. *Personality and Individual Differences*, 125:30–37, 2018. ISSN 0191-8869. doi: 10.1016/j.paid.2017.12.023.

11. Aaron L. Pincus, Emily B. Ansell, Claudia A. Pimentel, Nicole M. Cain, Aidan G. C. Wright, and Kenneth N. Levy. Initial construction and validation of the pathological narcissism inventory. *Psychological Assessment*, 21(3):365–379, 2009. ISSN 1040-3590. doi: 10.1037/a0016530.

12. Andrea Fossati, Antonella Somma, Serena Borroni, Kristian E. Markon, and Robert F. Krueger. The personality inventory for dsm-5 brief form: Evidence for reliability and construct validity in a sample of community-dwelling italian adolescents. *Assessment*, 24 (5):615–631, 2015. ISSN 1552-3489. doi: 10.1177/1073191115621793.

13. Judith A. Feeney, Patricia Noller, and Mary Hanrahan. Assessing adult attachment. *Advances in Personal Relationships*, 5:128–152, 1994.

14. Andrea Fossati, Judith A. Feeney, Deborah Donati, Michela Donini, Liliana Novella, Maria Bagnato, Elena Acquarini, and Cesare Maffei. On the dimensionality of the attachment style questionnaire in italian clinical and nonclinical participants. *Journal of Social and Personal Relationships*, 20(1):55–79, 2003. ISSN 1460-3608. doi: 10.1177/02654075030201003.
