# Supplementary material for: Leave the world(view) behind, but keep the words: The effect of conspiracism on writing
Source: PLoS One. 2026 Apr 29;21(4):e0346496. doi: 10.1371/journal.pone.0346496 (PMC13127973; doi:10.1371/journal.pone.0346496)
Supplement: S2 File — (PDF) [file pone.0346496.s002.pdf]

Inclusivity in global research

Ethical considerations, permits and authorship

This section is applicable to all research types.

Provide details as to who granted permissions and/or consent for the study to take place in the Methods section of your manuscript. This should include the names of **all** ethics boards, governmental organizations, community leaders or other bodies that provided approval for the study. If individuals provided approval refer to these people by their role or title but do not list their name(s).

- The study was preregistered and ethical approval was obtained from the Ethical Committee of Roma Tre University (Italy) in February 2024.

If there were any deviations from the study protocol after approval was obtained please provide details of these changes in the Methods section of your manuscript.

- No deviations from the approved study protocol occurred after approval was obtained.

Did this study involve local collaborators that are residents of the country where the research was conducted or members of the community studied? If you do not have any authors from said communities, please provide an explanation for this below.

- No. The study was conducted entirely by a single institutional research group, and no local collaborators or members of the studied community were involved as authors or formal collaborators.

Everyone listed as an author should meet PLOS' criteria for authorship and all individuals who meet these criteria should be included in the author byline, rather than the acknowledgements. For further information please see the journal's Authorship Policy.

Human subjects research (e.g. health research, medical research, cross-cultural psychology)

Did you obtain written informed consent from a representative of the local community or region before the research took place? How did you establish who speaks for the community? Details of written informed consent obtained from study participants should be reported separately in the Methods section of your manuscript.

- The study was conducted among university students who participated on a voluntary and anonymous basis and received course credit as compensation. Participants were assigned an identification code upon entering the classroom. All participants provided written informed consent.

How did members of the local community provide input on the aims of the research investigation, its methodology, and its anticipated outcome(s)?

- Members of the local community did not provide direct input into the aims, methodology, or anticipated outcomes of the study. The research was designed and conducted entirely by the research team, and community members were involved solely as voluntary study participants.

When engaging with the local community, how did you ensure that the informed consent documents and other materials could be understood by local stakeholders?

- All informed consent documents and study materials were written in clear, accessible language appropriate for the target population. As the study was conducted among university students, the materials were designed to be easily understandable by individuals with a university-level educational background. Participants were given the opportunity to ask questions before providing written informed consent.

Will the findings of the research be made available in an understandable format to stakeholders in the community where the study was conducted (e.g. via a presentation, summary report, copies of publications, etc.)? Please provide details of how this will be achieved.

- The findings of the study will be made available through academic publications. As the study was conducted in an academic setting with university students, participants and other interested stakeholders will be able to access the published results upon request. The results will be presented in a clear and understandable format consistent with standard scientific dissemination practices.

Non-human subjects research using specimens/ animals collected as part of the study, or those housed in archival collections. Examples include archaeology, paleontology, botany and zoology.

Did the permission you obtained from a local authority to perform the study include an agreement on access to outputs and benefit sharing? This may include procedures to enable fair distribution of the benefits and resources arising from the research performed. Please include any details of Prior Informed Consent and Benefit Sharing Agreements obtained. These may be required by field-specific regulations, for example the Convention on Biological Diversity (CBD) and the associated Nagoya Protocol.

N/A

If the material used in your study was imported, please A) provide the year it was imported and B) indicate whether permits were obtained to import/export the materials used, C) provide details of any permits obtained. If this information is not available, please indicate this.

N/A

If you used archival specimens, please state how the material used in your study was acquired by the institute it is held in and provide details of any permits obtained for the original excavations/ sample collection. If this information is not available, please indicate this.

N/A

How was the potential cultural significance of the materials collected in your study to local communities considered in your research design? Were Indigenous peoples and/or local researchers and institutions involved with archaeological excavations / collection of specimens? If so, please provide a description of their involvement.

N/A

If your manuscript includes photographs of human remains please indicate whether authors obtained permission from descendants or affiliated cultural communities to do so.

N/A
