# Supplementary material for: Leave the world(view) behind, but keep the words: The effect of conspiracism on writing
Source: PLoS One. 2026 Apr 29;21(4):e0346496. doi: 10.1371/journal.pone.0346496 (PMC13127973; doi:10.1371/journal.pone.0346496)
Supplement: Attachment — Submitted filename: R2R_JuliaRoberts_r1 2.pdf [file pone.0346496.s003.pdf]

Response to Reviewers
manuscript: PONE-D-25-17874

We thank the four reviewers for carefully reading our manuscript and for providing their constructive comments. In the following, our replies to the reviewers' comments (e.g., Comment R1:1) include «verbatim excerpts» from the revised manuscript, together with their corresponding updated Section or line numbers (e.g., Section "Other psychological contributors", Line 603).

Reviewer #1

This is a well-done paper that attempts to answer an important question. Further, no one else, as far as I am aware, have attempted to do this. I support moving towards publication.

Thank you.

- **[Comment R1:1]** Ln. 2 I am not sure that conspiracy theories flourish under uncertainty. The beliefs seem to be pretty stable over time at the mass and individuals levels. So, I am not sure this sentence is needed. The question you really want to address is how these narratives start and who starts them.

  Thank you. We removed the statement about uncertainty from the abstract and reinforced the point that conspiracy beliefs are relatively stable over time (see Line 24). In the second paragraph (Line 40), we clarified our objectives: we explicitly mentioned *conspiracism* as the main hypothesized predictor for the emergence of conspiracy narratives and explained the underlying mechanisms by which it operates.

  [Line 24] «Such narratives are widely endorsed (Klofstad et al., 2024; Oliver & Wood, 2014), persist across historical periods (Brotherton, 2016; Uscinski & Parent, 2014) and are relatively stable (Uscinski et al., 2022; Williams et al., 2024).»

  [Line 40] «Here, we tackle the question as to how conspiracy narratives emerge. We focus on *conspiracism*, a worldview marked by pervasive distrust of authorities and powerful actors (Bowes, Costello, & Tasimi, 2023; Bruder, Haffke, Neave, Nouripanah, & Imhoff, 2013; Imhoff & Bruder, 2013; Imhoff & Lamberty, 2018) that predisposes individuals to interpret and believe events as the outcome of a conspiracy (Bruder et al., 2013; Dagnall, Drinkwater, Parker, Denovan, & Parton, 2015; Imhoff & Bruder, 2013). Reliance on conspiratorial explanations has been associated with fundamental psychological motives, including the epistemic need for understanding and certainty, the existential drive for control and safety, and the social desire to maintain positive personal or group identities (Biddlestone et al., 2025; Douglas & Sutton, 2023; Douglas, Sutton, & Cichocka, 2017; Douglas et al., 2019). Individuals confronting uncertainty or threat frequently adopt conspiratorial explanations to

mitigate feelings of unpredictability and vulnerability (van Prooijen, 2020; van Prooijen & Jostmann, 2012). Individuals high in conspiracism--characterized by low tolerance for ambiguity (Biddlestone et al., 2025; Marchlewska, Cichocka, & Kossowska, 2018), a pronounced tendency to jump to conclusions (Kuhn, Lieb, Freeman, Andreou, & Zander-Schellenberg, 2021), and heightened sensitivity to perceiving meaningful connections among random events (Müller & Hartmann, 2023; van Prooijen, Douglas, & De Inocencio, 2018; van der Wal, Sutton, Lange, & Braga, 2018)--actively seek hidden motives and ultimate truths to make sense of what happens around them (Bangerter, Wagner-Egger, & Delouvée, 2020; Franks, Bangerter, & Bauer, 2013). They also frequently attribute intentionality (Brotherton & French, 2015) and agency to their environment (Douglas, Sutton, Callan, Dawtry, & Harvey, 2016; van der Tempel & Alcock, 2015). Consequently, they are prone to build implausible associations and inferences, identifying conspiracies even where none exist (van Prooijen & van Vugt, 2018).»

- **[Comment R1:2]** I think the paper's intent is a good one - I have yet to see a paper attempt to see if people generated conspiracy theories in response to something. Obviously, people do make up conspiracy theories, they are man made things. Somebody has to make them up. I would like to see the authors set this up more clearly: what are the alternative mechanisms in play? People can make them up or what? They are only made up by insincere people or by people with serious reasoning deficiencies?

  We do know, at least anecdotally, that people do come to conspiracy theories on their own intuition. Consider the all of the ancillary conspiracy theories created in the QAnon community. People in that group were making up new things everyday and everyone seemed to have their own set of conspiracy theories.

  This gets me to the treatment: This treatment was a Hollywood movie and not a real event. So, I wonder what this really tells us. Having seen the movie, it is weird and its not clear what the cause of the apocalypse is.

  I would agree with the authors that it may take more than a conspiracy worldview to create conspiracy theories. It may take some bits of schizotypy and traits like that.

In the revised manuscript, we now state more clearly that our primary mechanism of interest is a top-down, worldview-driven inference process (Line 137), where conspiracism drives the interpretation and reconstruction of ambiguous events. Because our data did not provide strong support for this mechanism, we now discuss more explicitly alternative mechanisms by which conspiracy narratives may emerge. In particular, we added a new section in the Discussion (see "Other psychological contributors" from Line 1142) in which we consider additional psychological traits potentially responsible for the generation of conspiracy narratives beyond conspiracism. These are: schizotypy (Line 1148), magical thinking (Line 1161), errors in probabilistic reasoning (Line 1175), belief in specific CTs (Line 1186), and motivated cognition (Line 1206).

We updated the part of the Discussion dealing with our cumulative hypothesis (see "Cumulative effect" from Line 1223). We emphasize that individual contributions to conspiratorial discourse may be small, but that fully fledged CTs can emerge cumulatively from elaborations within communities (e.g., QAnon).

Finally, regarding the treatment (i.e., the Hollywood movie), we clarified in the manuscript why we chose this ambiguous fictional scenario; see our reply to Comment R3:1 and Comment R3:2 (Line 148 and Line 905 in the manuscript).

[Line 137] «We hypothesize a link between conspiracism and the tendency to interpret and reconstruct ambiguous information as a result of a conspiracy. Specifically, we propose that conspiracy narratives result from top-down cognitive processes, where pre-existing beliefs and expectations guide how individuals interpret ambiguous events. We argue that cognitive biases typically associated with conspiracism lead individuals to impose conspiratorial interpretations onto ambiguous situations, particularly when epistemic needs are salient (e.g., interpreting an ambiguous

situation). As a consequence, these individuals reconstruct complex or uncertain information to align it with their overarching conspiratorial frameworks.»

[Line 1223] «As our data appear to suggest, the actual impact of conspiracism on the emergence of conspiratorial narratives may be relatively small and, as discussed above, contingent on other traits and motivations. Given that CTs do exist, how do they originate and propagate? It is possible that individual contributions to conspiratorial discourse might be modest, yet fully fledged CTs could emerge cumulatively (Koper, 2024). Initial ideas may gradually develop into elaborated narratives through repeated social interactions among multiple individuals over time (Klein, Clutton, & Polito, 2018; Schatto-Eckrodt, Clever, & Frischlich, 2024; Tangherlini, Shahsavari, Shahbazi, Ebrahimzadeh, & Roychowdhury, 2020; Weigmann, 2018). The QAnon movement illustrates this process: it encapsulates many specific beliefs without a single official version (Enders et al., 2022), is continually molded to accommodate satellite narratives, including mutually conflicting ones (Bromley & Richardson, 2023), and thus functions as a dynamic ecosystem in which ancillary narratives are generated, recombined, and personalized. Each believer assembles their own conspiratorial "package." Even before QAnon, analyses of the subreddit r/conspiracy similarly suggested a decentralized, cumulative pattern of narrative construction (Klein et al., 2018). Future research could investigate this cumulative hypothesis by employing paradigms inspired by cultural transmission studies (Bangerter, 2000; Stubbersfield, Tehrani, & Flynn, 2018), allowing researchers to examine how narratives evolve and transform across repeated social exchanges.»

- **[Comment R1:3]** Try to consistent with the language. Pick your terms, define them, and stick with them. I see conspiracism, conspiracy worldview, and conspiracy thinking. Are these the same thing?

  Thank you very much. The previous version of the manuscript was too loose and sometimes inconsistent in its terminology. We have reworked it completely and have taken great care to be consistent and clear in our terminology.

  We now commit to use *conspiracism* as an umbrella term (see Line 43) and reduced unnecessary variation in wording (e.g., switching between *conspiracy worldview*, *conspiracy thinking*). We refer to more specific constructs (e.g., *conspiracy mentality*, *belief in CTs*) only when such distinction is crucial (see Line 1186).

  [Line 43] «Note that the this is a deliberate simplification. *Conspiracism* has been described and operationalized in multiple ways, including conspiracist worldview (Dagnall et al., 2015), monological belief system (Goertzel, 1994), generic conspiracist ideation (Brotherton, French, & Pickering, 2013), conspiracy mentality (Bruder et al., 2013), conspiracy thinking (Uscinski & Parent, 2014), and conspiracist mindset (Nera, WagnerEgger, Bertin, Douglas, & Klein, 2021). Given the general and exploratory nature of this work, we use the term *conspiracism* (see e.g., (Barkun, 2013; Enders et al., 2024; Miani, Cruz, & Lewandowsky, 2025)) to indicate both the general tendency to believe in CTs *and* the possession of a conspiracy worldview. Such distinction, however, is important and will be discussed in the limitations.»

  [Line 1186] «Another potential predictor of the emergence of conspiratorial narratives is belief in specific CTs. Although conceptually related, correlated, and often used interchangeably by researchers (Nera, 2024), belief in CTs and conspiracy mentality are distinct psychological constructs (Biddlestone et al., 2025; Imhoff, Bertlich, & Frenken, 2022; Nera, 2024; Sutton, Douglas, & Trella, 2024; Trella, Sutton, & Douglas, 2024). Conspiracy mentality reflects a general tendency to distrust authority and suspect hidden motives, typically assessed with abstract or non-specific statements, and may be more closely associated with general skepticism, political cynicism, and

information-seeking motives. Conversely, belief in specific CTs involves endorsement of concrete and often epistemically risky claims and is more strongly linked to pathological cognitive tendencies, such as magical thinking, schizotypy, or reduced analytic reasoning (Sutton & Douglas, 2020; Trella et al., 2024). Given their more concrete and assertive nature, specific CTs may represent a stronger predictor than conspiracy mentality for the generation of conspiratorial narratives, which typically require elaboration, detail, and a degree of belief commitment that aligns more naturally with endorsement of specific, risky claims than with generalized skepticism alone. Our effect sizes support this conjecture: the relationship between the essays' level of conspiratorial narrative and conspiracism was higher for the more epistemically risky generic conspiracist scale (Brotherton et al., 2013) compared to the conspiracy mentality scale (Stojanov & Halberstadt, 2019).»

- **[Comment R1:4]** The monological discussion, lines 86-94, could be removed. Monological belief systems and conspiracy mentality/worldview, are not the same thing. Both would show correlations between conspiracy theory beliefs, but the underlying mechanisms would be different. In the former, beliefs drive beliefs; in the latter a latent disposition drives beliefs.

  Yes, we have removed the mention of monological belief systems as it was not well placed. We have also substantially revised the section on language (see "The Language of Conspiracy Theories" from Line 60), which now focuses primarily on research that directly links language use to conspiracism.

- **[Comment R1:5]** While LOCO is a great database and tool, it stretches the definition of conspiracy theory a bit. Bigfoot and Elvis are included in that database, but the existence of bigfoot is not a conspiracy theory (who is conspiring?) and a singer faking his own death (where is the malicious intent or harm?) would not fit most definitions. They are epistemically-suspect beliefs, but they do not allege a conspiracy, necessarily.

  In accordance with the suggestion of reviewer #3 (see Comment R3:3), we have added a subsection in the limitations where we discuss the limitations of both LOCO and DONALD (see "LOCO and DONALD's limitations" from Line 1101). In this section, we explicitly acknowledge that some LOCO seed events stretch common definitions of conspiracy theories and do not necessarily involve maliciousness (see Line 1113).

  [Line 1113] «Second, LOCO was built around events that are known to have generated CTs, gathered from surveys measuring belief in specific CTs (Douglas & Sutton, 2011; Jensen, 2013). However, it could be argued that not all of these events can be unequivocally considered CTs. For example, there is nothing inherently harmful about Bigfoot itself (Uscinski, 2025), or about the (alleged) faked deaths of popular figures such as Elvis Presley and Princess Diana (or Paul McCartney). Although these events can be considered as facts allegedly known by authorities but hidden from the public (consistent with the definition proposed by Lantian, Muller, Nurra, and Douglas (2016)), they seem to lack the clear malicious intent that is often taken to characterize CTs (Douglas et al., 2019; Nera & Schöpfer, 2023). This ambiguity in the seed events may limit the content validity of LOCO as a corpus of genuine conspiratorial documents and, by extension, of the dictionary derived from it.»

- **[Comment R1:6]** I think the interpretation of the results on line 301-305 is correct. The conspiracy mentality construct is just a general disposition, and in itself might not get a person to make up conspiracy theories all the time without other psychological motives involved.

  Indeed, see our reply to Comment R1:3 (Line 1186), where we consider the more epistemically risky "belief in specific CTs". Thank you.

Reviewer #2

Using AI-driven classification and dictionary-based lexical analysis, this manuscript presents an innovative exploration of whether an individual's conspiratorial worldviews primes the spontaneous production of conspiratorial narratives when interpreting ambiguous stimuli. While the authors do not find a correlation between conspiratorial worldview and narrative structure, they do find linguistic correlates of conspiracy beliefs in the narratives produced by participants; further, they provide an intriguing interpretation of these results in the argument that they may be evidence that the individuals producing conspiracy theories in online discourse may simply have mastered the conventions of the genre rather than being true believers. Despite these strengths, I believe the manuscript would benefit from revision, particularly with respect to the methodological framing of the paper.

Thank you.

- **[Comment R2:1]** ChatGPT is used both to develop the prompt asking participants to producing a narrative interpretation of "Leave the World Behind" and to classify the resulting narratives in the discussion starting on p. 6; while the prompt itself is strong, ChatGPT is known to rely heavily on coherence and wellformedness, which, as the authors themselves note, means that it may miss covert conspiratorial content. Deschrijver (2021) discusses the metapragmatic and metacommunicative nature of conspiracy theories, and may provide useful insight into ways to further refine the instructions given to ChatGPT to help it capture some of the more covert or implicit features of conspiracy theories.

  Additionally, the black box nature of ChatGPT's scoring introduces a potential issue for replication, despite the validation steps provided in the study. The authors might consider adding in an additional layer of manual coding, particularly for texts with low cohesion or unconventional structure, or might note this as a limitation of the study.

  We thank the reviewer for these insightful comments that prompted us to do a new set of analyses. We now explicitly acknowledge that LLMs seem to rely on narrative coherence and stylistic cues (Line 467). To address this, we conducted additional analyses testing the extent to which our LLM-based assessments were moderated by indices of textual quality (e.g., cohesion, syntactic and lexical sophistication). These results, reported in the Results section (Line 766), allow us to quantify how strongly coherence and related features influence LLM assessments of conspiratorial content.

  In line with the suggestion to capture more covert or implicit aspects of conspiracy narratives, we introduced an additional LLM classification round based on multiple dimensions (e.g., deception, social impact), drawing on prior work (Deschrijver, 2021; Douglas et al., 2019). Rather than relying on a single conspiracy score, this new prompt asks the model to evaluate several theoretically grounded dimensions of conspiratorial discourse. We briefly describe these additional analyses in the Results (Line 721) and provide full details in the Appendix (Section "An alternative evaluation using ChatGPT", Line 1318).

  Regarding the black-box nature and replicability of LLM-based scoring, unfortunately, we do not have funds nor workforce to add an additional manual coding. However, we added a dedicated subsection on LLM-related limitations (see "LLMs' classification" from Line 1067), where we discuss: (1) the absence of explicit anchors in our prompt, (2) the impact of textual quality on LLM judgments, (3) the possibility of conspiratorial bias in the LLMs, and (4) broader concerns about the *"documentation debt"* (see reply to Comment R3:3).

  Finally, it is important to stress that in this revision we compared ChatGPT with Claude (Anthropic) against the human-coded ground-truth and found that Claude showed slightly higher agreement. We therefore relied on Claude for our main LLM-based assessments, while reporting this choice as a deviation from our preregistration (Line 303). For transparency, we also provide the previous correlation between ChatGPT scores and conspiracism (Line 721).

[Line 467] «Second, because there is some evidence that LLMs rely on narrative coherence and stylistic cues (Su, Zhuo, Mansurov, Wang, & Nakov, 2023; Wu & Aji, 2025) these measures serve also to assess LLM's evaluations, and whether they are sensitive to textual quality.»

[Line 766] «To evaluate whether the textual quality of essays could affect the LLM's assessments of conspiratorial levels (see e.g., (Su et al., 2023; Wu & Aji, 2025)), we conducted a series of regressions predicting essays' conspiratorial scores from participants' conspiracism, adding textual quality as moderator. A significant interaction could indicate that the relationship between participants' conspiracism and their essays is moderated by the quality of the texts. Note that because we preregistered the use of ChatGPT, but diverged from the preregistration because we discovered that Claude performed better, we performed these tests for both ChatGPT and Claude along with dictionary. We ran nine regression models in which we predict the essay conspiratorial scores (evaluated with dictionary, chatGPT, and Claude) from conspiracism interacting with measures of textual quality (syntactic sophistication, lexical sophistication, and cohesion). Overall, conspiracy narratives evaluated via LLMs were consistently and positively associated with textual qualities (βs > .10, ps < .056) but not with conspiracism (|βs| < .04, ps > .05). When essays' conspiracy level was assessed via dictionary, the reverse pattern was found, with a positive and consistent association with conspiracism (βs > .17, ps < .001) but not with textual quality, which in fact showed a negative association ($\beta = -12$, $p < .024$). As for the interactions, on average, the absolute coefficients of the models for LLM-based assessments were larger than those for the dictionary-based assessment, although none were significant at $p < .05$, suggesting a higher impact of textual quality on LLM's assessments of conspiratorial narratives. Overall, this suggests that the dictionary-based scoring was less affected by textual quality compared to LLM's scores (a thorough description of the methods with results is reported in the Appendix D).»

[Line 721] «As we mentioned, reliance on Claude (vs ChatGPT) represents a deviation from our preregistration. For transparency, we report the correlation between ChatGPT assessment and conspiracism level: $r_{383} = .01$, $[-.09, .11]$, $p = .821$. Note that we also tested an alternative prompt to gather conspiracy scores from LLMs (see Appendix C for details). Instead of using the definition of Nera and Schöpfer (2023), we prompted Claude to rate in each essay different dimensions theoretically associated with conspiracism (e.g., deception, social relevance, etc, (Deschrijver, 2021; Douglas et al., 2019)). Scores following this new prompt were positively associated with S2 based on the definition of Nera and Schöpfer (2023) ($r_{383} = .67$, $[.62, .72]$, $p < .001$), but they were not associated with conspiracism ($r_{383} = -.01$, $[-.11, .09]$, $p = .9$).»

[Line 1067] «A first limitation concerns the prompt we used to elicit LLM judgments, which did not include explicit anchors. Unlike our lexical sophistication norms, where we provided concrete exemplars (e.g., *mum* vs *neurotic*), here neither the models nor human raters were given examples illustrating different levels of conspiratorial strength. In the absence of such anchors, it is difficult to calibrate what counts as a *"very strong conspiratorial narrative"*. Future work relying on LLMs' classification could test whether adding explicit anchors or benchmark texts improves the reliability and validity of these assessments. A second limitation is inherent to the classification method itself. LLMs may reliably detect conspiratorial narratives primarily when they appear in clearly structured and coherent texts. If models are more effective when narratives are cohesive, they may fail to identify conspiratorial content expressed in fragmented, tangential, or loosely organized ways. This interpretation is indirectly supported by our exploratory analyses, which indicated that LLM-based assessments were moderated by indices of textual quality. By contrast, a simpler dictionary measure based on word counts successfully captured the relationship between conspiracism and conspiratorial vocabulary use, suggesting that LLM classifications may be more

sensitive to narrative coherence than to conspiratorial content alone. Beyond these issues, there are additional concerns about LLMs' behavior when assessing conspiratorial content. An additional concern is that LLMs can themselves exhibit a degree of conspiratorial bias (Corso, Pierri, & Morales, 2025). If a model treats conspiratorial claims as relatively plausible, an essay containing such content might receive a lower conspiratorial rating than warranted, resulting in a biased assessment. Finally, there is a broader concern of replicability. Many LLMs are trained on large proprietary corpora whose exact composition is undisclosed, contributing to the *"documentation debt"* (Bandy & Vincent, 2021) that complicates replication and comparability across studies. Replicability is further constrained because we cannot share participants' essays due to restrictions imposed by the Roma Tre ethics committee. Nevertheless, we report the prompts verbatim, specify the model used, and provide a repository with validation materials alongside human ratings, thereby facilitating partial reproducibility and future extensions.»

[Line 303] «To establish a human-coded benchmark, we randomly selected 180 documents spanning the entire range of ChatGPT scores, which were independently evaluated by three native Italian speakers (60 documents per rater) using the same scoring method. We compared ChatGPT ratings (using the gpt-4o-mini model) with those of another LLM, Claude by Anthropic (using the claude-3-5-sonnet model). The correlation between human ratings and LLMs's ratings was overall robust, indicating strong agreement, although Claude ($r_{176} = .701$, $[.617, .769]$, $p < .001$) performed better than ChatGPT ($r_{178} = .676$, $[.588, .748]$, $p < .001$).»

- **[Comment R2:2]** The black box nature of ChatGPT is again relevant to the generation of Age of Acquisition (AoA) norms on p. 13. While the authors explain that their use of generative AI here is due to the small pool of existing AoA norms for Italian and provide validation measures for the generated data against the existing dataset, this approach divorces language from speakers and it is unclear how ChatGPT came to those ratings. Łuniewska et al. (2016) find consistency in AoA ratings from a cross-linguistic perspective across different language families - it would be interesting to know how ChatGPT's ratings compare to data collected from native speakers of languages related to Italian to further support the validation of this approach.

We have rewritten the description of how we generated lexical sophistication norms. In the previous version, we placed too much emphasis on AoA itself, whereas our primary goal was to obtain a measure of lexical sophistication. We now clarify that the prompt to ChatGPT was primarily designed to elicit perceived lexical sophistication, with AoA used only as a conceptual anchor (see Section "Lexical Sophistication", Line 603).

To address the concerns about LLM-generated norms and the link between norms and actual speakers, we now explicitly report convergent validity not only for Italian, but also for English and German (Line 625). We also explicitly acknowledge that the cognitive mechanisms underlying these LLM-generated norms remain opaque and that further work is needed.

[Line 603] «To our knowledge, no existing word norms directly quantify lexical sophistication. Instead, researchers have often inferred lexical sophistication indirectly via metrics such as processing time that is known to correlate with age of acquisition (AoA; (Kuperman, Stadthagen-Gonzalez, & Brysbaert, 2012)), word frequency (Brysbaert & New, 2009), and word length (Lewis & Frank, 2016). To address this gap, we generated lexical sophistication norms using LLMs, which have shown promising results in approximating human psycholinguistic judgments (Green, KONG, Brysbaert, & Keogh, 2025). Specifically, we extracted a list of Italian lemmas and their part-of-speech tags from the IRMA corpus (Carrella, Miani, & Lewandowsky, 2023), and prompted ChatGPT to estimate the complexity of each word analogously to AoA ratings. We prompted ChatGPT to assess how sophisticated a word would appear to a native Italian speaker (see S5). We anchored the ratings in AoA norms because they reflect the developmental trajectory of lexical acquisition, where early-acquired words (e.g., *mum*, *food*) are typically perceived as less complex, and later-acquired

or specialized terms (e.g., *syphilis*, *kerosene*, *neurotic*) as more complex. AoA norms have previously been used as a proxy for linguistic sophistication (Kim, Crossley, & Kyle, 2017) and have captured sophistication in nominal compounds in conspiracy texts (Miani, van der Plas, & Bangerter, 2024). We obtained sophistication ratings for 190,507 Italian lemmas. These ratings showed strong convergent validity with the existing Italian AoA dataset (Montefinese, Vinson, Vigliocco, & Ambrosini, 2019), yielding a correlation of $r = .64$ [.611, .665], $p < .001$ across the 1,912 overlapping lemmas.»

[Line 625] «Using the same prompt, we also generated complexity norms for English and German (each with $N > 100,000$ lemmas). These norms similarly correlated well with established AoA datasets (in English: $r = .74$, based on 28,000 words from (Kuperman et al., 2012); in German: $r = .71$, based on 2,958 words from (Birchenough, Davies, & Connelly, 2016)). [Footnote: While these correlations are encouraging, the cognitive mechanisms underlying LLM-generated norms remain opaque. Future work should explore whether these norms exhibit the same cross-linguistic consistency as human AoA ratings (Łuniewska et al., 2015).] Applying the English LLM-based norms to the LOCO corpus, we found that conspiracy texts exhibited significantly higher lexical sophistication than mainstream texts ($\beta = .07$, [.06, .09], $SE = .01$, $t = 9.76$, $p < .001$). [Footnote: Analyses were conducted using multilevel regression with a random intercept for $k = 200$ topics.]»

- **[Comment R2:3]** In measuring syntactic sophistication (p. 12), the authors don't seem to consider the contributions of semantics or specific verb type. Counterfactuals and hypotheticals are often salient structures in conspiracy theories, as are negatively evaluative verbs (Catenaccio, 2022), and adding these considerations into the measure of syntactic sophistication may further enrich these findings.

  Thanks. We acknowledged the work of Catenaccio (2022) and added the following remark:

  [Line 566] «Note that beyond syntactic and lexical sophistication, previous research has also examined how semantic and pragmatic features (e.g., counterfactuals, hypotheticals, and evaluative language) contribute to conspiracy discourse, particularly in relation to refutational reasoning (Catenaccio, 2022). While these aspects fall outside the scope of our structurally focused analysis, we acknowledge their relevance and encourage future work to incorporate verb semantics and discourse-level patterns for a more comprehensive understanding of conspiratorial rhetoric.»

- **[Comment R2:4]** This paper tackles an important and underexplored question: how do conspiratorial worldviews shape the linguistic construction of narratives? The multi-method approach and large sample size are commendable. However, the analysis would benefit from more engagement with metapragmatic dimensions of discourse and from additional methodological considerations. Addressing these issues would significantly strengthen the paper's contribution to both conspiracy theory research and the linguistics of belief construction.

  Thanks!

Reviewer #3

I see great merit in this study in general, as I agree with the authors that much more research must be done on the rhetoric and content of conspiracy theories rather than looking at psychological priors associated with conspiracism alone. I would like to see some version of this article find its way to publication. That said, I do have a few reservations about the essay in its current form:

- **[Comment R3:1]** 1. Can you provide the rationale for selecting Leave the World Behind and for structuring this study around a fictional film? While Sam Esmail's Mr. Robot series and this film both hint toward fictional conspiracies, neither film purports to represent actual events. A more intuitive film might be a documentary about an unexplained actual phenomenon or event that has yet to surface in existing conspiracy theory discourse. I am also concerned that the film was produced for an Anglophone and specifically American audience, but the respondents in the study would have viewed the film with Italian subtitles presumably. Translation itself is an act of interpretation. Thus, both the original cultural and language context of the film introduce additional variables given that the study was conducted on Italian participants. The authors acknowledge the challenge in moving between conspiratorial discourse in different languages, but they do not really address how the film's original production might pose problems for their study.

  Thank you. In the revised manuscript, we now provide a much clearer rationale for our choice of stmilus (i.e., the film *Leave the World Behind*) and for building the study around a fictional film. We also directly address the issue of language and cultural context (see Line 187 and Subsection "The film" from Line 148).

  > [Line 148] «To test this hypothesis, participants watched the apocalyptic psychological thriller *Leave the World Behind* (Esmail, 2023) and subsequently were prompted to write an essay interpreting its meaning. This film was chosen specifically for its psychological ambiguity and the open-ended conclusion fostering uncertainty that could potentially trigger interpretations consistent with conspiracism (van Prooijen & Jostmann, 2012). Therefore, we measured participants' conspiracism, their essays' level of conspiracy narrative, and tested whether the two are positively associated.»

  > [Line 187] «Participants viewed the film *Leave the World Behind* (Esmail, 2023) and subsequently completed an essay-writing task in which they were prompted to interpret the film by providing their personal understanding and meaning (see S1).
  > *Leave the World Behind* is not overtly conspiratorial; a conspiratorial reading is only one among several possible interpretations afforded by its narrative. We deliberately chose a non-explicitly conspiratorial film because our aim was not to test the persuasiveness of conspiratorial argumentation (see, e.g., (Adornetti et al., 2025)), but to examine whether people high in conspiracy mentality "fill the gaps" of an ambiguous situation, in this case, a fictional narrative.
  > The film is deliberately open-ended: it features unexplained phenomena, a fractured narrative timeline, and an unresolved ending that invite viewers to infer what is happening "behind the scenes". Prompting an interpretation of an overtly conspiratorial film would likely have constrained essays to a similar conspiratorial baseline. What interested us instead were the *additional* narrative and interpretative elements emerging from top-down cognitive processes, where pre-existing beliefs and expectations guide how individuals interpret ambiguous events.
  > In fact, our hypothesis is that individuals with a conspiratorial predisposition would be more likely to fill these narrative gaps with conspiratorial explanations, partly because of their higher need for structure and certainty and lower tolerance for ambiguity (Biddlestone et al., 2025; Marchlewska et al., 2018; van Prooijen, 2020; van Prooijen & Jostmann, 2012). In this sense, the film serves as a useful tool for observing how people project conspiratorial meanings onto an ambiguous story even in the absence of an explicitly conspiratorial storyline.
  > Participants viewed the film dubbed in Italian without subtitles. Italian dubbing is professionally standardized and ubiquitous. For most Italians a dubbed Hollywood film feels as natural as a domestic one (Antonini & Chiaro, 2009). Italians have been steeped in US screen culture since the 1950s, and recent streaming has only deepened that familiarity. Themes such as institutional breakdown, technological fragility, and post-pandemic anxiety resonate well beyond the US context. Decades of media-globalization research (Straubhaar, 1991) show that such cultural proximity allows viewers to appropriate foreign narratives without losing interpretive nuance.»

- **[Comment R3:2]** 2. The prompt, as the authors correctly acknowledge, asked the participants to interpret the film. Given that it is a film set in a fictional dystopic future, it seems likely the participants will not deploy conspiracy theory

narratives as they know it's a work of art rather than an account of actual events. There are so many possible modes of interpretation—analogical, allegorical, literal, or symptomatic—that could be deployed that may not seem explicitly conspiratorial but that may surface in conspiratorial rhetoric in a different way. Therefore, I am not convinced that prompt would easily yield a response with conspiratorial rhetoric. Perhaps the authors could bolster their case for this address these concerns.

Thank you for raising this concern. We now explicitly address it in the Discussion by considering alternative modes of interpretation that participants may have adopted when responding to our prompt (see Line 842 and Line 853).

[Line 842] «Our goal, however, was not to influence or measure participants' attitudes, but rather to observe their sense-making activity. Fictional narratives are routinely used as vehicles for commenting on real-world institutions and power relations (Martin, 2018; Yazell, Petersen, Marx, & Fessenbecker, 2021). As such, regardless of the the perceived realism of the film, conspiratorial interpretations can arise at a literal or allegorical level, or through analogies with what participants perceive as the real world. Thus, if conspiracism shapes interpretive habits, it should remain detectable in essays about a fictional scenario, even though the openness of the task also allowed for non-conspiratorial readings and may therefore have lowered the base rate of explicitly conspiratorial narratives.»

[Line 853] «Nevertheless, it is also possible that participants interpreted the film as a work of art rather than an account of actual or possible events. In this case, they may have understood the instructions as an invitation to provide a film-criticism-style response, focusing on the director's intentions and overarching message instead of the narrative content per se.»

- **[Comment R3:3]** 3. LOCO corpus and ChatGPT: I also have reservations about using the LOCO corpus and ChatGPT to perform genre and narrative classification. An LLM, as the authors also acknowledge, is best primed to detect the presence of conspiracy narrative already in circulation. In terms of the LOCO corpus, the article could be strengthened by addressing some of the problems with this corpus and conspiracy theory classification raised in Mompelat et al.'s "How 'Loco' is the LOCO Corpus? Annotating the Language of Conspiracy Theories." Although likely less applicable now, it would also be worthwhile to address the concerns with the way ChatGPT was initially trained on corpora that might miss the mark when it comes to correctly identifying certain genres raised in Bandy and Vincent's "Addressing 'Documentation Debt' in Machine Learning Research." Precisely because it's not always clear how these LLMs reach their conclusions, I am wary of relying upon them as a way of classifying narratives they previously haven't encountered. Thus, this study may say more about the limitations of using LLMs for detecting novel conspiracy theories than it does about the inclination of participants predisposed to conspiracism to craft new conspiracy theory narratives to make sense of ambiguous phenomena or events.

Thanks. In the revised manuscript, we now explicitly discuss the limitations of both the LOCO and DONALD corpora (see Section "LOCO and DONALD's limitations", from Line 1101). See also our reply to Comment R2:1 (Section "LLMs' classification", from Line 1067).

We acknowledge LOCO's limitation as suggested by Mompelat et al. (2022); see Line 1126. We expand our discussion of the "black box" nature of LLMs and recognize the documentation debt (Section LLMs' classification, from Line 1067, see also reply to Comment R2:1).

[Line 1126] «Third, beyond content validity, not all documents in LOCO actually contain CTs, with a portion of documents that mention the event's keyword (e.g., "Sandy Hook") without any conspiratorial discussion of the event itself (the Sandy Hook school shooting) (Mompelat et al., 2022). This reflects a well-known problem in corpus collection (Diab, Nefriana, & Lin, 2024), namely a trade-off

`between sample size and accuracy. On the one hand, gathering all documents from a source known to deliver CTs ensures a large sample size but increases the likelihood of false positives, as it also includes documents that do not mention CTs (Mompelat et al., 2022). On the other hand, false positives are minimized if documents are carefully selected to include CTs, but this results in a costly and time-consuming endeavour, with limited sample size and heterogeneity, and may introduce sample bias if documents are selected based on the same lexical features that are later used to analyze their lexical content. These corpus-level constraints should be kept in mind when interpreting the performance and generalizability of both the dictionary and the measures derived from it.»`

- **[Comment R3:4]** I did, however, find the article's findings when it came to the use of conspiracy-related vocabulary or syntactic sophistication to be sound because this outcome is not dependent on a particular corpus, LLM, or genre. The authors might consider dispensing with the conspiratorial narrative findings at this stage or qualifying those claims even further, as this part of the article undercuts its strengths.

  Thank you, we agree. We conducted supplemental analyses to identify a potential source of measurement error (i.e., why the LLM did not perform as expected), while acknowledging that it is also possible the texts contained no genuinely conspiratorial narratives (i.e., reflecting participants' lack of motivation). These are limitations that future research should investigate systematically.

Reviewer #4

This paper examines an important question—do conspiracy believers spontaneously generate conspiracy narratives when interpreting threatening, uncertain events?—using a highly original method: an analysis of essays discussing the meaning of a movie. The methods, while innovative (e.g., using ChatGPT to rate the conspiracist nature of essays), were subjected to multiple checks to ensure their validity (e.g., having human judges rate the conspiracist nature of the essays and examining how their ratings correlate with ChatGPT scores). The authors also provide interesting additional analyses related to syntactic structure and vocabulary. I am enthusiastic about this research. I have moderate comments that should be addressed before I can recommend acceptance.

Thank you.

- **[Comment R4:1]** Moderate comments: The discussion could be improved. As it stands, it is quite long and somewhat scattered across various interpretations of "why didn't it work?". My personal view on null results is that one can always find numerous reasons why an experimental manipulation did not yield the expected outcome. I believe the discussion should focus on theoretically relevant points, particularly the discrepancy between conspiracist narrative ratings (as coherent pieces) and conspiracist vocabulary. I was struck by the weak correlation between these two variables. From my naïve perspective, conspiracist vocabulary should be strongly related to whether a text actually argues for the existence of a conspiracy. This discrepancy deserves deeper exploration and opens up very rich and exciting theoretical questions.

  Thanks. We agree that the discussion is rather long. At the same time, given the novelty of linking conspiracism to spontaneous written output, we believe it is important to provide a detailed discussion of methodological and interpretative limitations, as these can help guide future research building on this first attempt. Indeed, one open question (assuming the LLM is accurate) is why conspiracism was associated with conspiratorial lexicon but not with conspiratorial narrative: perhaps low (vs high) cognitive effort of using a "readily available" vocabulary (vs building a coherent narrative; see Subsection "Cognitive demand", from Line 929)? However, we cannot rule out

the possibility that the LLM did not reliably identify conspiracy narratives (see our reply to Comment R2:1). We also added possible interpretations and expanded the section on potential psychological contributors beyond conspiracism (see our reply to Comment R1:2).

- **[Comment R4:2]** In the introduction, you tie conspiracism to the need-based approach to conspiracy theories proposed by Douglas et al. (2017) (line 41). However, these are actually two different concepts. Douglas et al.'s (2017) article discusses conspiracy belief and belief in conspiracy theories, and does not mention conspiracy mindset, conspiracism, or conspiracy mentality. Please revise the phrasing in this paragraph to avoid implying that the need-based approach is central to understanding the generic conspiracist mindset.

  Thanks, we have adjusted the need-based statement (Line 48).

  [Line 48] «Note that the need-based approach, advanced by Douglas et al. (2017, 2019), was initially related to belief in CTs. There is however research that has linked these needs also to conspiracism (meta-analyzed in (Biddlestone et al., 2025)), although the strength slightly varies with the way conspiracy belief is measured.»

- **[Comment R4:3]** While I understand that it does not affect the results, I find the decision to aggregate the two dimensions of the Conspiracy Mentality Scale in Study 1 into a single score questionable. This scale is notable for its attempt to disentangle conspiracist ideation from "healthy suspicion" toward authorities. This issue becomes especially relevant in the discussion between Studies 1 and 2, where some CMS items are described as reflecting an accurate perception of the Italian political system. These items actually belong to the "healthy skepticism" subscale, so it is unsurprising that they do not appear strongly conspiracist. I therefore recommend conducting analyses that include both subdimensions separately (e.g., CT_narrative ∼ healthy_skepticism + conspiracist_ideation) in Study 1. The intermediary discussion should be updated accordingly.

  We conducted the recommended additional analyses for both studies, treating the relevant subdimensions separately (see Line 335 and Line 412). We also extended the correlation tables (Table S4 and Table S5) to report correlations between the essay-based measures (including conspiratorial narrative and conspiratorial lexicon) and each subdimension of the conspiracy scales used in the two studies.

  [Line 335] «Since the CMS has two-factor structure, we also ran an additional regression treating the two CMS subdimensions (conspiratorial ideation and healthy skepticism) as separate predictors of the conspiratorial narrative scores. Again, there were no significant effects of either subscale (ideation: $\beta = .05$, $[-.08, .19]$, $SE = .07$, $t = .76$, $p = .446$; skepticism: $\beta = .02$, $[-.12, .15]$, $SE = .07$, $t = .23$, $p = .818$), indicating that the null finding observed in our original analysis does not depend on the aggregation of the two subcomponents of the CMS.»

  [Line 412] «As in Study 1, we ran an additional regression treating the five subdimensions of the GCB as separate predictors of the conspiratorial narrative scores. Again, there were no significant effects of individual subscale except one (Government malfeasance: $\beta = .31$, $[.04, .58]$, $SE = .14$, $t = 2.25$, $p = .027$; Malevolent global conspiracies: $\beta = -.04$, $[-.29, .21]$, $SE = .13$, $t = -.31$, $p = .754$; Extraterrestrial cover-up: $\beta = -.24$, $[-.47, -.02]$, $SE = .11$, $t = -2.15$, $p = .034$; Personal wellbeing: $\beta = .10$, $[-.18, .39]$, $SE = .14$, $t = .72$, $p = .473$; Control of information: $\beta = -.05$, $[-.31, .21]$, $SE = .13$, $t = -.38$, $p = .705$).»

- **[Comment R4:4]** Minor comment:
  The abstract begins with the sentence "Conspiracy theories (CTs) tend to flourish under uncertainty." This phrasing suggests that you manipulated uncertainty, whereas the study examined whether individuals spontaneously mobilize

conspiracy narratives to explain ambiguous stories. Consider removing or rephrasing this sentence.

I wish the authors the best of luck with their work.

Kenzo Nera (signed review)

Thanks. In line with Comment R1:1, we have removed it.

References

Adornetti, I., Altavilla, D., Chiera, A., Deriu, V., Gerna, A., Picca, L., & Ferretti, F. (2025). Testing the persuasiveness of conspiracy theories: a comparison of narrative and argumentative strategies. *Cognitive Processing*. doi: 10.1007/s10339-025-01282-9

Antonini, R., & Chiaro, D. (2009). The perception of dubbing by italian audiences. In *Audiovisual translation* (pp. 97–114). Palgrave Macmillan UK. doi: 10.1057/9780230234581_8

Bandy, J., & Vincent, N. (2021). Addressing "documentation debt" in machine learning: A retrospective datasheet for bookcorpus. In J. Vanschoren & S. Yeung (Eds.), *Proceedings of the neural information processing systems track on datasets and benchmarks* (Vol. 1). Retrieved from `https://datasets-benchmarks-proceedings.neurips.cc/paper_files/paper/2021/file/54229abfcfa5649e7003b83dd4755294-Paper-round1.pdf`

Bangerter, A. (2000). Transformation between scientific and social representations of conception: The method of serial reproduction. *British Journal of Social Psychology*, *39*(4), 521–535. doi: 10.1348/014466600164615

Bangerter, A., Wagner-Egger, P., & Delouvée, S. (2020). How Conspiracy Theories Spread. In M. Butter & P. Knight (Eds.), *Routledge Handbook of Conspiracy Theories* (First ed., pp. 206–218). Abingdon, Oxon ; New York, NY : Routledge, 2020.: Routledge. doi: 10.4324/9780429452734-2_5

Barkun, M. (2013). *A culture of conspiracy: Apocalyptic visions in contemporary america* (2nd ed.). University of California Press.

Biddlestone, M., Green, R., Douglas, K. M., Azevedo, F., Sutton, R. M., & Cichocka, A. (2025). Reasons to believe: A systematic review and meta-analytic synthesis of the motives associated with conspiracy beliefs. *Psychological Bulletin*, *151*(1), 48–87. doi: 10.1037/bul0000463

Birchenough, J. M. H., Davies, R., & Connelly, V. (2016). Rated age-of-acquisition norms for over 3, 200 german words. *Behavior Research Methods*, *49*(2), 484–501. doi: 10.3758/s13428-016-0718-0

Bowes, S. M., Costello, T. H., & Tasimi, A. (2023). The conspiratorial mind: A meta-analytic review of motivational and personological correlates. *Psychological Bulletin*, *149*(5–6), 259–293. doi: 10.1037/bul0000392

Bromley, D. G., & Richardson, J. T. (2023). The qanon conspiracy narrative: Understanding the social construction of danger. In *The social science of qanon* (pp. 159–175). Cambridge University Press. doi: 10.1017/9781009052061.014

Brotherton, R. (2016). *Suspicious minds: Why we believe conspiracy theories* (Paperback edition ed.). New York London: Bloomsbury Sigma.

Brotherton, R., & French, C. C. (2015). Intention Seekers: Conspiracist Ideation and Biased Attributions of Intentionality. *PLOS ONE*, *10*(5), e0124125. doi: 10.1371/journal.pone.0124125

Brotherton, R., French, C. C., & Pickering, A. D. (2013). Measuring Belief in Conspiracy Theories: The Generic Conspiracist Beliefs Scale. *Frontiers in Psychology*, *4*. doi: 10.3389/fpsyg.2013.00279

Bruder, M., Haffke, P., Neave, N., Nouripanah, N., & Imhoff, R. (2013). Measuring individual differences in generic beliefs in conspiracy theories across cultures: Conspiracy mentality questionnaire. *Frontiers in Psychology*, *4*(April), 225. doi: 10.3389/fpsyg.2013.00225

Brysbaert, M., & New, B. (2009). Moving beyond Kučera and Francis: A critical evaluation of current word frequency norms and the introduction of a new and improved word frequency measure for American English. *Behavior Research Methods*, *41*(4), 977–990. doi: 10.3758/BRM.41.4.977

Carrella, F., Miani, A., & Lewandowsky, S. (2023). IRMA: the 335-million-word Italian coRpus for studying MisinformAtion. In A. Vlachos & I. Augenstein (Eds.), *Proceedings of the 17th conference of the european chapter of the association for computational linguistics* (pp. 2339–2349). Dubrovnik, Croatia: Association for Computational Linguistics. doi: 10.18653/v1/2023.eacl-main.171

Catenaccio, P. (2022). A corpus-driven exploration of conspiracy theorising as a discourse type: Lexical indicators of argumentative patterning. In *Conspiracy theory discourses* (pp. 25–48). John Benjamins Publishing Company. doi: 10.1075/dapsac.98.02cat

Corso, F., Pierri, F., & Morales, G. D. F. (2025). Do androids dream of unseen puppeteers? probing for a conspiracy mindset in large language models.
doi: 10.48550/ARXIV.2511.03699

Dagnall, N., Drinkwater, K., Parker, A., Denovan, A., & Parton, M. (2015). Conspiracy theory and cognitive style: a worldview. *Frontiers in Psychology*, *6*. doi: 10.3389/fpsyg.2015.00206

Deschrijver, C. (2021). On the metapragmatics of conspiracy theory: Scepticism and epistemological debates in online conspiracy comments. *Journal of Pragmatics*, *182*, 310–321. doi: 10.1016/j.pragma.2021.02.010

Diab, A., Nefriana, R., & Lin, Y.-R. (2024). Classifying conspiratorial narratives at scale: False alarms and erroneous connections. *Proceedings of the International AAAI Conference on Web and Social Media*, *18*, 340–353. doi: 10.1609/icwsm.v18i1.31318

Douglas, K. M., & Sutton, R. M. (2011). Does it take one to know one? endorsement of conspiracy theories is influenced by personal willingness to conspire. *British Journal of Social Psychology*, *50*(3), 544–552. doi: 10.1111/j.2044-8309.2010.02018.x

Douglas, K. M., & Sutton, R. M. (2023). What are conspiracy theories? a definitional approach to their correlates, consequences, and communication. *Annual Review of Psychology*, *74*(1), 271–298. doi: 10.1146/annurev-psych-032420-031329

Douglas, K. M., Sutton, R. M., Callan, M. J., Dawtry, R. J., & Harvey, A. J. (2016). Someone is pulling the strings: Hypersensitive agency detection and belief in conspiracy theories. *Thinking & Reasoning*, *22*(1), 57–77. doi: 10.1080/13546783.2015.1051586

Douglas, K. M., Sutton, R. M., & Cichocka, A. (2017). The psychology of conspiracy theories. *Current Directions in Psychological Science*, *26*(6), 538–542. (PMID: 29276345) doi: 10.1177/0963721417718261

Douglas, K. M., Uscinski, J. E., Sutton, R. M., Cichocka, A., Nefes, T., Ang, C. S., & Deravi, F. (2019). Understanding conspiracy theories. *Political Psychology*, *40*(S1), 3–35. doi: 10.1111/pops.12568

Enders, A., Klofstad, C., Littrell, S., Miller, J., Theocharis, Y., Uscinski, J. E., & Zilinsky, J. (2024). Leftright political orientations are not systematically related to conspiracism. *Political Psychology*. doi: 10.1111/pops.13017

Enders, A., Uscinski, J. E., Klofstad, C. A., Wuchty, S., Seelig, M. I., Funchion, J. R., ... Stoler, J. (2022). Who supports qanon? a case study in political extremism. *The Journal of Politics*, *84*(3), 1844–1849. doi: 10.1086/717850

Esmail, S. (2023). *Leave the world behind.* (Film, Netflix)

Franks, B., Bangerter, A., & Bauer, M. W. (2013). Conspiracy theories as quasi-religious mentality: an integrated account from cognitive science, social representations theory, and frame theory. *Frontiers in Psychology*, *4*. ([Online; accessed 2022-07-04]) doi: 10.3389/fpsyg.2013.00424

Goertzel, T. (1994). Belief in conspiracy theories. *Political Psychology*, *15*(4), 731. doi: 10.2307/3791630

Green, C., KONG, A. P. H., Brysbaert, M., & Keogh, K. (2025). Crowdsourced and ai-generated age of acquisition (aoa) norms for vocabulary in print: Extending the kuperman et al. (2012) norms. doi: 10.31234/osf.io/698mw_v2

Imhoff, R., Bertlich, T., & Frenken, M. (2022). Tearing apart the “evil” twins: A general conspiracy mentality is not the same as specific conspiracy beliefs. *Current Opinion in Psychology*, *46*, 101349. doi: 10.1016/j.copsyc.2022.101349

Imhoff, R., & Bruder, M. (2013). Speaking (Un-)Truth to Power: Conspiracy Mentality as a Generalised Political Attitude. *European Journal of Personality*, *28*(1), 25–43. doi: 10.1002/per.1930

Imhoff, R., & Lamberty, P. (2018). How paranoid are conspiracy believers? toward a more finegrained understanding of the connect and disconnect between paranoia and belief in conspiracy theories. *European Journal of Social Psychology*, *48*(7), 909–926. doi: 10.1002/ejsp.2494

Jensen, T. (2013). *Democrats and Republicans differ on conspiracy theory beliefs - Public Policy Polling — publicpolicypolling.com.* https://www.publicpolicypolling.com/polls/democrats-and-republicans-differ-on-conspiracy-theory-beliefs/.

Kim, M., Crossley, S. A., & Kyle, K. (2017). Lexical sophistication as a multidimensional phenomenon: Relations to second language lexical proficiency, development, and writing quality. *The Modern Language Journal*, *102*(1), 120–141. doi: 10.1111/modl.12447

Klein, C., Clutton, P., & Polito, V. (2018). Topic Modeling Reveals Distinct Interests within an Online Conspiracy Forum. *Frontiers in Psychology*, *9*. doi: 10.3389/fpsyg.2018.00189

Klofstad, C., Christley, O., Diekman, A., Enders, A., Funchion, J., Hemm, A., ... Uscinski, J. E. (2024). The new satanic panic. *Political Science Quarterly*, *140*(2), 249–268. doi: 10.1093/psquar/qqae081

Koper, A. J. (2024). More than a theory: Conspiracy theorising as a practical tradition. *Political Studies Review*, *23*(4), 994–1010. doi: 10.1177/14789299241292573

Kuhn, S. A. K., Lieb, R., Freeman, D., Andreou, C., & Zander-Schellenberg, T. (2021). Coronavirus conspiracy beliefs in the german-speaking general population: endorsement rates and links to reasoning biases and paranoia. *Psychological Medicine*, *52*(16), 4162–4176. doi: 10.1017/s0033291721001124

Kuperman, V., Stadthagen-Gonzalez, H., & Brysbaert, M. (2012). Age-of-acquisition ratings for 30,000 English words. *Behavior Research Methods*, *44*(4), 978–990. doi: 10.3758/s13428-012-0210-4

Lantian, A., Muller, D., Nurra, C., & Douglas, K. M. (2016). Measuring belief in conspiracy theories: Validation of a French and English single-item scale. *International Review of Social Psychology*, *29*(1), 1–14.

Lewis, M. L., & Frank, M. C. (2016). The length of words reflects their conceptual complexity. *Cognition*, *153*, 182–195. doi: 10.1016/j.cognition.2016.04.003

Łuniewska, M., Haman, E., Armon-Lotem, S., Etenkowski, B., Southwood, F., Anđelković, D., ... Ünal-Logacev, Ö. (2015). Ratings of age of acquisition of 299 words across 25 languages: Is there a cross-linguistic order of words? *Behavior Research Methods*, *48*(3), 1154–1177. doi: 10.3758/s13428-015-0636-6

Marchlewska, M., Cichocka, A., & Kossowska, M. (2018). Addicted to answers: Need for cognitive closure and the endorsement of conspiracy beliefs. *European Journal of Social Psychology*. doi: 10.1002/ejsp.2308

Martin, C. J. (2018). Imagine all the people: Literature, society, and cross-national variation in education systems. *World Politics*, *70*(3), 398–442. doi: 10.1017/s0043887118000023

Miani, A., Cruz, N., & Lewandowsky, S. (2025). Still very much dead and alive: Incoherence in conspiracism as a departure from bayesian rationality. Retrieved from `https://osf.io/preprints/osf/t6a54_v3` doi: 10.31219/osf.io/t6a54_v3

Miani, A., van der Plas, L., & Bangerter, A. (2024). Loose and tight: Creative formation but rigid use of nominal compounds in conspiracist texts. *The Journal of Creative Behavior*, *58*(1), 114–127. doi: 10.1002/jocb.633

Mompelat, L., Tian, Z., Kessler, A., Luettgen, M., Rajanala, A., Kübler, S., & Seelig, M. (2022). How “loco” is the loco corpus? annotating the language of conspiracy theories. In *Proceedings of the 16th linguistic annotation workshop (law-xvi) within lrec2022.*

Montefinese, M., Vinson, D., Vigliocco, G., & Ambrosini, E. (2019). Italian age of acquisition norms for a large set of words (itaoa). *Frontiers in Psychology*, *10*. doi: 10.3389/fpsyg.2019.00278

Müller, P., & Hartmann, M. (2023). Linking paranormal and conspiracy beliefs to illusory pattern perception through signal detection theory. *Scientific Reports*, *13*(1). doi: 10.1038/s41598-023-36230-0

Nera, K. (2024). Analyzing the causation between conspiracy mentality and belief in conspiracy theories: Potential pitfalls and leads to address them. *Zeitschrift für Psychologie*, *232*(1), 44–49. doi: 10.1027/2151-2604/a000533

Nera, K., & Schöpfer, C. (2023). What is so special about conspiracy theories? Conceptually distinguishing beliefs in conspiracy theories from conspiracy beliefs in psychological research. *Theory & Psychology*, *33*(3), 287–305. doi: 10.1177/09593543231155891

Nera, K., WagnerEgger, P., Bertin, P., Douglas, K. M., & Klein, O. (2021). A powerchallenging theory of society, or a conservative mindset? upward and downward conspiracy theories as ideologically distinct beliefs. *European Journal of Social Psychology*, *51*(45), 740–757. doi: 10.1002/ejsp.2769

Oliver, J. E., & Wood, T. (2014). Medical conspiracy theories and health behaviors in the united states. *JAMA Internal Medicine*, *174*(5), 817. doi: 10.1001/jamainternmed.2014.190

Schatto-Eckrodt, T., Clever, L., & Frischlich, L. (2024). The seed of doubt: Examining the role of alternative social and news media for the birth of a conspiracy theory. *Social Science Computer Review*, *42*(5), 1160–1180. doi: 10.1177/08944393241246281

Stojanov, A., & Halberstadt, J. (2019). The Conspiracy Mentality Scale: Distinguishing Between Irrational and Rational Suspicion. *Social Psychology*, *50*(4), 215–232. doi: 10.1027/1864-9335/a000381

Straubhaar, J. D. (1991). Beyond media imperialism: Assymetrical interdependence and cultural proximity. *Critical Studies in Mass Communication*, *8*(1), 39–59. doi: 10.1080/15295039109366779

Stubbersfield, J., Tehrani, J., & Flynn, E. (2018). Faking the news: Intentional guided variation reflects cognitive biases in transmission chains without recall. *Cultural Science Journal*, *10*(1), 54–65. doi: 10.5334/csci.109

Su, J., Zhuo, T. Y., Mansurov, J., Wang, D., & Nakov, P. (2023). *Fake news detectors are biased against texts generated by large language models.* arXiv. doi: 10.48550/ARXIV.2309.08674

Sutton, R. M., & Douglas, K. M. (2020). Conspiracy theories and the conspiracy mindset: implications for political ideology. *Current Opinion in Behavioral Sciences*, *34*, 118–122. doi: 10.1016/j.cobeha.2020.02.015

Sutton, R. M., Douglas, K. M., & Trella, C. (2024). Conspiracy mentality versus belief in conspiracy theories: Response to nera and some recommendations for researchers. *Zeitschrift für Psychologie*, *232*(1), 50–54. doi: 10.1027/2151-2604/a000549

Tangherlini, T. R., Shahsavari, S., Shahbazi, B., Ebrahimzadeh, E., & Roychowdhury, V. (2020). An automated pipeline for the discovery of conspiracy and conspiracy theory narrative frameworks: Bridgegate, Pizzagate and storytelling on the web. *PLOS ONE*, *15*(6), e0233879. doi: 10.1371/journal.pone.0233879

Trella, C., Sutton, R. M., & Douglas, K. M. (2024). Semantic and causal relations between the conspiracy mentality and belief in conspiracy theories. *Zeitschrift für Psychologie*, *232*(1), 717. Retrieved from `http://dx.doi.org/10.1027/2151-2604/a000545` doi: 10.1027/2151-2604/a000545

Uscinski, J. E. (2025). Defining conspiracy theory and related terms. In *Conceptualizing extreme beliefs and behaviors* (pp. 139–155). Oxford University PressNew York, NY. doi: 10.1093/9780197760222.003.0008

Uscinski, J. E., Enders, A., Klofstad, C., Seelig, M., Drochon, H., Premaratne, K., & Murthi, M. (2022). Have beliefs in conspiracy theories increased over time? *PLOS ONE*, *17*(7), e0270429. doi: 10.1371/journal.pone.0270429

Uscinski, J. E., & Parent, J. M. (2014). *American conspiracy theories.* Oxford University Press. doi: 10.1093/acprof:oso/9780199351800.001.0001

van Prooijen, J.-W. (2020). An Existential Threat Model of Conspiracy Theories. *European Psychologist*, *25*(1), 16–25. doi: 10.1027/1016-9040/a000381

van Prooijen, J.-W., Douglas, K. M., & De Inocencio, C. (2018). Connecting the dots: Illusory pattern perception predicts belief in conspiracies and the supernatural: Illusory pattern perception. *European Journal of Social Psychology*, *48*(3), 320–335. doi: 10.1002/ejsp.2331

van Prooijen, J.-W., & Jostmann, N. B. (2012). Belief in conspiracy theories: The influence of uncertainty and perceived morality. *European Journal of Social Psychology*, *43*(1), 109–115. doi: 10.1002/ejsp.1922

van Prooijen, J.-W., & van Vugt, M. (2018). Conspiracy Theories: Evolved Functions and Psychological Mechanisms. *Perspectives on Psychological Science*, *13*(6), 770–788. doi: 10.1177/1745691618774270

van der Tempel, J., & Alcock, J. E. (2015). Relationships between conspiracy mentality, hyperactive agency detection, and schizotypy: Supernatural forces at work? *Personality and Individual Differences*, *82*, 136–141. doi: 10.1016/j.paid.2015.03.010

van der Wal, R. C., Sutton, R. M., Lange, J., & Braga, J. P. N. (2018). Suspicious binds: Conspiracy thinking and tenuous perceptions of causal connections between co-occurring and spuriously correlated events. *European Journal of Social Psychology*, *48*(7), 970–989. doi: 10.1002/ejsp.2507

Weigmann, K. (2018). The genesis of a conspiracy theory: Why do people believe in scientific conspiracy theories and how do they spread? *EMBO reports*, *19*(4). doi: 10.15252/embr.201845935

Williams, M. N., Ling, M., Kerr, J. R., Hill, S. R., Marques, M. D., Mawson, H., & Clarke, E. J. R. (2024). People do change their beliefs about conspiracy theoriesbut not often. *Scientific Reports*, *14*(1). doi: 10.1038/s41598-024-51653-z

Wu, M., & Aji, A. F. (2025). Style over substance: Evaluation biases for large language models. In O. Rambow, L. Wanner, M. Apidianaki, H. Al-Khalifa, B. D. Eugenio, & S. Schockaert (Eds.), *Proceedings of the 31st international conference on computational linguistics* (pp. 297–312). Abu Dhabi, UAE: Association for Computational Linguistics. Retrieved from `https://aclanthology.org/2025.coling-main.21/`

Yazell, B., Petersen, K., Marx, P., & Fessenbecker, P. (2021). The role of literary fiction in facilitating social science research. *Humanities and Social Sciences Communications*, *8*(1). doi: 10.1057/s41599-021-00939-y
