# Supplementary material for: Leave the world(view) behind, but keep the words: The effect of conspiracism on writing
Source: PLoS One. 2026 Apr 29;21(4):e0346496. doi: 10.1371/journal.pone.0346496 (PMC13127973; doi:10.1371/journal.pone.0346496)
Supplement: Attachment — Submitted filename: 2026 02 18 - R2R_JuliaRoberts_r2.pdf [file pone.0346496.s004.pdf]

Response to Reviewers
manuscript: `PONE-D-25-17874R1`

We thank the editor and Dr. Kenzo Nera for reading the manuscript carefully and for identifying inconsistencies and oversights.

Editor

This manuscript raises an interesting question. Please address the reviewers' comments. Additionally, there appears to be a slight misalignment between the title and the main findings of the paper. The title suggests an effect of conspiracism on writing, yet the results from the three studies diverge somewhat from this focus. Please clarify the connections and distinctions between related terms such as writing, conspiratorial narratives, conspiratorial discourse, and genuine conspiratorial beliefs.

We thank the Editor for highlighting the need to clarify the conceptual distinctions among related terms.

Regarding the distinction between narratives and discourse, we acknowledge that narratology traditionally distinguishes between *story* (the content of events), *discourse* or *plot* (their textual organization), and *narration* (the enunciative act that produces them). This distinction is extensively discussed in the theoretical literature (e.g., Chatman, 1980; Genette, 1972). However, these differences are not central to the aims of the present work. Our analyses do not focus on formal narratological structure, but rather on the presence of conspiracy-related content and linguistic features in participants' written interpretations. For the sake of clarity and concision, and because our theoretical framework does not hinge on these structural distinctions, we therefore use the terms *conspiratorial narratives* and *conspiratorial discourse* largely interchangeably throughout the manuscript. We have now clarified this point in the revised version of the text. We added a footnote when reviewing the literature on the language of conspiracy theories:

> [Line 71] «Narratological theories traditionally distinguish between story (the content of events), discourse or plot (their textual organization), and narration (the enunciative act that produces them; see e.g., (Chatman, 1980; Genette, 1972)). However, these distinctions are not central to the present investigation, because our analyses focus on the presence of conspiracy-related content and linguistic features rather than on formal narrative structure. Therefore, the terms *conspiratorial narratives* and *conspiratorial discourse* are used largely interchangeably throughout this article for the sake of clarity and concision.»

Regarding *genuine belief*, thank you. We have clarified the phrasing throughout the text. We replaced "*genuine belief*" with "*genuinely held beliefs*" to distinguish between strategically produced conspiratorial claims and those that reflect sincerely endorsed convictions.

We also rewrote the abstract to reduce any potential ambiguity in our terminology and to make clear that we *did* find an effect of conspiracism on writing. Unexpectedly, this effect was not the emergence of a conspiratorial narrative, but rather the use of conspiracy-related vocabulary.

> [Line 2] «We investigate whether a stable predisposition to interpret events as the result of conspiracies--conspiracism--is associated with the spontaneous generation of conspiratorial content in writing when interpreting ambiguous information. Across two studies ($N = 385$), participants watched the apocalyptic thriller *Leave the World Behind* and wrote an essay interpreting its meaning. Each essay was rated by a Large Language Model for its conspiratorial narrative content, that is, the degree to which the text contains claims that the public is being pervasively lied to about aspects of reality, enabling some groups to enact a harmful, self-serving agenda. Contrary to our preregistered hypothesis, we did not find an association between participants' conspiracism and their essays' level of conspiratorial narrative content. Exploratory linguistic analyses revealed that conspiracism was associated with greater use of conspiracy-related vocabulary, a disproportionate use of sophisticated words, and increased syntactic complexity. These results suggest that conspiracism may emerge more readily at the lexical level rather than through fully structured narratives. We discuss potential methodological and theoretical factors contributing to these unexpected results, including the roles of context, perceived relevance, motivation, and collective social dynamics. We also consider the possibility that conspiracism may not directly translate into conspiratorial narratives. If so, we recommend comparative research on online vs offline conspiratorial writing to clarify whether conspiracy theories emerge spontaneously from genuine beliefs or are constructed strategically, detached from genuinely held beliefs.»

Reviewer #1

As far as I am concerned, I am satisfied with how the authors addressed my initial comments. At this point, I only have minor comments:

Thank you.

- **[Comment R1:1]** P4: "we focus on one potential candidate, namely conspiracism, and test whether it is capable of shaping the interpretation and communication of narratives." This suggests a causal impact of conspiracism on narrative generation. The method does not enable causal conclusions, so consider rephrasing ("we examine if conspiracism is associated with..." (I know it's a cliché and annoying comment, but I think it is important)).

  Thanks, we changed both the abstract (see Line 2) and the main text (Line 134).

  [Line 2] «We investigate whether a stable predisposition to interpret events as the result of conspiracies--conspiracism--is associated to the spontaneous generation of conspiratorial narratives when interpreting ambiguous information.»

  [Line 134] «Here, we examine if *conspiracism* is associated with the interpretation and communication of narratives.»

- **[Comment R1:2]** P8: "although Claude (r176 = .701, [.617, .769], p < .001) performed better than ChatGPT (r178 = .676, [.588, .748], p < .001)" Since the confidence intervals overlap, I do not think you need to state that Claude performed better than ChatGPT. I'd just state that both LLMs did well.

  Revised

  [Line 314] «The correlation between human ratings and LLMs's ratings was overall robust, indicating strong agreement, although Claude ($r_{176} = .701$, $[.617, .769]$, $p < .001$) performed slightly better than ChatGPT ($r_{178} = .676$, $[.588, .748]$, $p < .001$; but note that the 95% confidence intervals overlap). Based on these validation steps, we concluded that LLMs provide reliable evaluations of conspiracism and that Claude performed slightly better than ChatGPT for Italian texts.»

- **[Comment R1:3]** p. 16: "being schizotypy the strongest psychopathological predictor of conspiracism" The grammar seems off here.

  Revised

  [Line 678] «Similar fragmented and associative thought patterns have been observed among individuals on the schizophrenia spectrum (Elvevåg, Foltz, Weinberger, & Goldberg, 2007; Paulsen et al., 1996; Willits, Rubin, Jones, Minor, & Lysaker, 2018), which partially overlaps with conspiracism (schizotypy is the strongest psychopathological predictor of conspiracism (Bowes, Costello, & Tasimi, 2023)).»

- **[Comment R1:4]** p. 18: "As for the interactions, on average, the absolute coefficients of the models for LLM-based assessments were larger than those for the dictionary-based assessment, although none were significant at p < .05, suggesting a higher impact of textual quality on LLMs' assessments of conspiratorial narratives."
  In the supplement, there is actually a significant interaction for graph D (the interaction is significant at p = .005). This might be difficult to interpret because the result with Claude is not significant (p = .14), but both are in the same direction, so there might be a meta-analytical effect here. It suggests that the conspiracy-ness of the text is negatively associated

with conspiracy mentality when the writing is of high quality, and positive when the writing is of poor quality. You mention this in the limitations.

Thank you for spotting this. We updated this section:

> [Line 792] «As for the interactions, on average, the absolute coefficients of the models for LLM-based assessments were larger than those for the dictionary-based assessment, although none (except one, see Model D in Table S3 and Figure S1) were significant at $p < .05$, suggesting an overall higher impact of textual quality on LLM's assessments of conspiratorial narratives.»

- **[Comment R1:5]** In the limitations:
  "A third additional concern is that LLMs can themselves exhibit a degree of conspiratorial bias [152]. If a model treats conspiratorial claims as relatively plausible, an essay containing such content might receive a lower conspiratorial rating than warranted, resulting in a biased assessment." I don't see this as a real problem, since the rating is continuous. Worst case, the average rating is a little high; it does not necessarily mean that it'll fuck up your results (except if it results in a ceiling effect that fails to capture high levels of conspiracism).

Good point, thanks. We added the following:

> [Line 1099] «Such bias, however, should be evenly distributed across the rating continuum and thus systematically decrease conspiratorial ratings for all texts. This would not be problematic unless an artificial floor effect is detected.»

- **[Comment R1:6]** I suppose it is in response to a reviewer, but I do not see the necessity to acknowledge that there might be other processes contributing to the emergence of conspiracy narratives (magical thinking, etc.), especially given the length of the limitations section. Of course, there are other processes involved; it's the same for every possible dependent variable in social psychology. That said, the "conspiracy as a cumulative work" hypothesis is really cool.
  Kenzo Nera (signed review)

Thanks!

References

Bowes, S. M., Costello, T. H., & Tasimi, A. (2023). The conspiratorial mind: A meta-analytic review of motivational and personological correlates. *Psychological Bulletin*, *149*(5–6), 259–293. doi: 10.1037/bul0000392

Chatman, S. B. (1980). *Story and discourse*. Cornell University Press.

Elvevåg, B., Foltz, P. W., Weinberger, D. R., & Goldberg, T. E. (2007). Quantifying incoherence in speech: An automated methodology and novel application to schizophrenia. *Schizophrenia Research*, *93*(1-3), 304–316. doi: 10.1016/j.schres.2007.03.001

Genette, G. (1972). *Figures III*. Editions du Seuil.

Paulsen, J. S., Romero, R., Chan, A., Davis, A. V., Heaton, R. K., & Jeste, D. V. (1996). Impairment of the semantic network in schizophrenia. *Psychiatry Research*, *63*(2-3), 109–121. doi: 10.1016/0165-1781(96)02901-0

Willits, J. A., Rubin, T., Jones, M. N., Minor, K. S., & Lysaker, P. H. (2018). Evidence of disturbances of deep levels of semantic cohesion within personal narratives in schizophrenia. *Schizophrenia Research*, *197*, 365–369. doi: 10.1016/j.schres.2017.11.014
